# Stress from cold and drought as drivers of functional trait spectra in North American angiosperm tree assemblages

**DOI:** 10.1002/ece3.3297

**Published:** 2017-08-14

**Authors:** Irena Šímová, Marta Rueda, Bradford A. Hawkins

**Affiliations:** ^1^ Center for Theoretical Study Charles University Praha Czech Republic; ^2^ Department of Ecology Faculty of Science Charles University Praha Czech Republic; ^3^ Department of Ecology & Evolutionary Biology University of California Irvine CA USA; ^4^ Department of Conservation Biology Estación Biológica de Doñana (EBD‐CSIC) Sevilla Spain

**Keywords:** cold tolerance, community assembly, environmental filtering, functional biogeography, macroecology, woody species

## Abstract

Understanding how environmental change alters the composition of plant assemblages, and how this in turn affects ecosystem functioning is a major challenge in the face of global climate change. Assuming that values of plant traits express species adaptations to the environment, the trait‐based approach is a promising way to achieve this goal. Nevertheless, how functional traits are related to species’ environmental tolerances and how trait spectra respond to broad‐scale environmental gradients remains largely unexplored. Here, we identify the main trait spectra for US angiosperm trees by testing hypotheses for the relationships between functional traits and species’ environmental tolerances to environmental stresses, as well as quantifying the environmental drivers of assemblage means and variances of these traits. We analyzed >74,000 community assemblages from the US Forest Inventory and Analysis using 12 functional traits, five traits expressing species’ environmental tolerances and 10 environmental variables. Results indicated that leaf traits, dispersal traits, and traits related to stem hydraulics were related to cold or drought tolerance, and their assemblage means were best explained by minimum temperatures. Assemblage means of traits related to shade tolerance (tree growth rate, leaf phosphorus content, and bark thickness) were best explained by aridity index. Surprisingly, aridity index, rather than minimum temperature, was the best predictors of assemblage variances of most traits, although these relationships were variable and weak overall. We conclude that temperature is likely to be the most important driver of functional community structure of North American angiosperm trees by selecting for optimum strategies along the cold and drought stress trade‐off. In turn, water availability primarily affects traits related to shade tolerance through its effect on forest canopy structure and vegetation openness.

## 1 INTRODUCTION

Documenting large‐scale patterns of plant form and function is necessary to understand how functional traits drive the response of species to the environment as well as how they mediate key ecosystem functions, such as carbon, nutrient, or water cycling (Lavorel & Garnier, 2002). A number of studies examining the large‐scale patterns of trait distributions have found strong relationships between trait values and environmental variables (e.g., Moles et al., 2009; Swenson & Weiser, 2010; Swenson et al., 2012), often interpreted as the effect of environment constraining species distributions via their traits. Nevertheless, the common assumption that traits corresponding to the broad‐scale environmental gradient are related to species’ tolerances to environmental conditions has rarely been tested (but see Stahl et al., 2013), probably due to the fact that measures of tolerances are more limited than trait values. Moreover, the majority of studies use a restricted number of plant functional traits such as height, specific leaf area, seed mass, or wood density (but see Moles et al., 2014), largely due to limited data availability. Although these commonly used traits represent key‐independent plant ecological strategies (Chave et al., 2009; Westoby, 1998; Wright et al., 2004), the extent to which they constrain species distributions along broad climatic gradients remains unclear (see, e.g., Laughlin, Fulé, Huffman, Crouse, & Laliberté, 2011; Stahl et al., 2013).

The main trade‐off in plant strategies can be viewed as a spectrum of adaptations to environmental stresses versus adaptations to disturbances (Lavorel & Garnier, 2002; Reich, 2014). Plants experiencing stressful conditions are typically characterized by slow tissue turnover, long lifespans, and traits that enhance resource conservation (Coley, 1988; Coley, Bryant, & Chapin, 1985). In contrast, relatively benign environmental conditions, as well as sufficient light and nutrient supply, can increase the intensity of competition selecting for traits related to fast growth, tissue turnover, and high potential for resource capture (Reich, 2014; Reich et al., 2003). The majority of plants, however, show characteristics of plant adaptations to stressful conditions (slow strategies, Reich 2014), although stress‐tolerant plants are variable in their ability to tolerate limiting factors (Niinemets & Valladares, 2006). There are several key stressors (environmental filters) that can presumably affect plant species distributions and community assemblages at large spatial scales. Exposure to low temperatures generally represents a major selective pressure (Hawkins et al, 2014; Zanne et al., 2014), although other stresses such as drought, nutrient availability, shade, and fire should be important as well (Bond & Keeley, 2005; Niinemets & Valladares, 2006; Ordoñez et al., 2009; Reich et al., 2003; Rueda, Godoy, & Hawkins, 2017a, 2017b; Stahl et al., 2013).

Here we aim to identify the trait spectra corresponding to the species’ environmental tolerances to stress and explore the environmental filters that constrain particular traits within these spectra. Specifically, we examine spatial variation in community assemblages across the conterminous USA using 17 tree traits. Twelve of them are considered “functional traits” (defined sensu Violle et al., 2007), including the most commonly used LHS traits (specific leaf area (L) height (H) and seed mass (S); Westoby, 1998). We additionally include five species’ environmental tolerances (nonfunctional traits; sensu Violle et al., 2007) representing tolerance to cold, drought, fire, water, and shade (see Table 1 and Appendix S1 for details). We extend the approach of Stahl et al. (2013) by including cold tolerance, likely the most important evolutionary adaptation for North American angiosperm trees (Hawkins et al., 2014; Latham & Ricklefs, 1993). To describe the spatial variation in community assemblages, we use both means and variances of each trait calculated per plot.

**Table 1:** The list of traits used in our analyses with units and explanation

Trait	Units	Explanation
SLA (Specific leaf area)	mm/mg	Leaf area/dry mass
Leaf N	% (log)	Leaf nitrogen content per leaf mass
Leaf P	% (log)	Leaf phosphorus content per leaf mass
Leaf shape	—	Leaf width/length
Seed mass	mg (log)	Seed weight
Dispersal mode	Categorical (1–3)	Animal/unassisted/wind
Wood density	mg/cm	Wood dry mass/volume
Growth rate	Ordinal (1–3)	Slow/moderate/fast
Lifespan	Ordinal (1–3)	Short/moderate/long
Height	m	Maximum tree height
Bark thickness	Ordinal (1–3)	Thin/moderately thick/thick
Winter buds size	mm	Longitude of winter buds
Cold tolerance	°C	The lowest temperature of species’ historical range.
Drought tolerance	Cardinal (1–5)	Physiological tolerance to water stress
Waterlogging tolerance	Cardinal (1–5)	Tolerance of reduced root‐zone soil oxygen availabilities
Shade tolerance	Cardinal (1–5)	The capacity for growth in the shade
Fire tolerance	Ordinal (1–4)	Ability to resprout or reestablish after fire

See Appendix S1 for a detailed description of the estimation of species’ environmental tolerances and Appendix S2 for trait values and sources.

To extend previous studies, we broadened the selection of environmental predictors to include 10 variables (nine measuring contemporary conditions and one historical). Although the most commonly used climatic variables such as temperature and precipitation have undoubtedly a strong effect on assemblage functional composition (Laughlin et al., 2011; Swenson et al., 2012), these variables alone do not capture all axes of environmental filters. We expect assemblage means of traits related to cold tolerance to be best explained by the gradient of minimum temperature across the conterminous USA. Traits related to drought and fire tolerance should be explained by summer precipitation. However, we also include other known drivers of water‐energy balance, namely maximum temperature of the warmest month, aridity index, solar radiation, soil moisture, and evapotranspiration, as they could be stronger predictors of these trait spectra (Larcher, 2003). Solar radiation as a variable related to light availability can be further associated with traits related to shade tolerance. Sites with sufficient water and temperature availability can suffer from nutrient limitation (Mayor, Wright, & Turner, 2014) affecting leaf traits or growth rate (Reich et al., 2003). Therefore, we also include a variable representing soil types. Soil moisture and soil types can further affect traits related to waterlogging tolerance, although these traits should be primarily affected by topography, here represented by elevation. To complicate the issue even more, species spatial distributions and resulting assemblage functional composition can be affected by historic events such as Pleistocene glaciation (Normand et al., 2011), a measure which we also include as a predictor.

Based on the extensive literature on trees, traits, and forest assemblages, we evaluate the following predictions. We expect dispersal traits to be primarily related to the species’ cold and drought tolerance as larger seeds should be favored under warm and wet conditions due to higher competitive pressure (Moles & Westoby, 2003). Seed size could be further related to shade tolerance as large seeds better establish in shaded conditions (Leishman & Westoby, 1994). Specific leaf area, leaf nutrient traits, whole plant growth, and mortality rate (or lifespan) are among the fundamental components of the slow‐fast trait spectra, and presumably they should be related to gradients of drought and nutrients as adaptations to these stresses involve resource maintenance (Coley et al., 1985; Reich et al., 2003; Wright et al., 2004). These traits could be further related to shade tolerance as shade‐tolerant species invest in resistant tissues in order to tolerate periods of low light, thus reflecting a “slow” strategy (Valladares & Niinemets, 2008).

Traits associated with stem hydraulics and the plant capacity to transport water such as maximum stem height and wood density should be primarily associated with plant drought tolerance (Chave et al., 2009; Preston, Cornwell, & DeNoyer, 2006; Ryan & Yoder, 1997). Besides drought tolerance, the values of traits associated with stem hydraulic could be further constrained by water excess (Lambers, Chapin, & Pons, 2008). A water supply trade‐off should be also reflected in leaf shape and associated vein density regulating whole plant transpiration (Nicotra et al., 2011). As bark protects stems from lethal heat, bark thickness should correlate with fire tolerance (Pausas, 2015). And finally, the ability of a tree to resist drought or shade should be reflected in the size of its winter buds (Sanz‐Pérez & Castro‐Díez, 2010).

According to the trait driver theory, assemblage trait variances should decrease with strong abiotic filtering (Enquist et al., 2015). We thus expect trait variances to be limited by the same environmental stressors that constrain the mean of a particular trait in an assemblage, subject to its general influence on communities. On the other hand, trait variances could also decrease with strong rates of competition (Enquist et al., 2015; Mayfield & Levine, 2010) that should be more intense in warm and wet environments.

## 2 METHODS

### 2.1 Forest data

The community data comprise 74,689 plots, 0.07 hectares each, in the contiguous USA extracted from the US Forest Service's Forest Inventory and Analysis database (http://www.fia.fs.fed.us/, accessed in January, 2012). The data used were collected between the years 2005 and 2010, corresponding to the recently updated 5‐year cyclical inventory. Only sites supporting at least two angiosperm tree species and coded as a “natural stand” were included in the analysis. Our study is restricted to angiosperms, as gymnosperms have very different evolutionary histories and have substantially different suites of traits by which they respond to stresses (Graham, 1999).

### 2.2 Traits and species’ environmental tolerances

Species‐level trait data were generated from numerous primary, secondary, and Internet sources for the 219 angiosperm species sampled by the FIA. Depending on the trait, values could be found from 81 to all species (Table S1 in Appendix S2). Twelve of the 17 traits and species’ environmental tolerances are continuous and five are ordinal or categorical (Table 1). Species names provided by the FIA and trait sources were updated and standardized using The Plant List (www.theplantlist.org). Note, the values of species’ environmental tolerances in most cases do not refer to direct measures of physiological tolerance of species but a realized tolerance directly measured at a site or, in case of the cold tolerance, estimated from the species’ historical range. Therefore, these values can be potentially affected by biotic interactions or by the human land use. Unfortunately, measures of physiological tolerances are not available for most of the species used in this study. Nevertheless, the probability that both physiological and realized tolerances are strongly correlated in North American trees is high (e.g., Hawkins et al., 2014).

To obtain community assemblage data with species trait information, we first generated a presence–absence matrix of the 219 angiosperm species across all sites. We then calculated a mean and variance value per plot site of each of the 17 traits (see Figs S1–S2 in Appendix S3 for correlation matrix for assemblage trait means and variances). These statistics were the response variables in statistical models of trait–environment associations.

### 2.3 Environmental predictors

Ten environmental variables were evaluated as potential filters, and values were generated for each FIA plot. Three climatic variables were extracted from the 30 arc‐sec Worldclim database (http://www.worldclim.org, collected during the period between 1960 and 1999): mean maximum temperature in the warmest month (“maximum temperature” hereafter), mean minimum temperature in the coldest month (“minimum temperature” hereafter), and summer precipitation. Additional potential environment drivers included the following: evapotranspiration, based on MODIS remote sensing data (Mu, Zhao, & Running, 2011), aridity index, calculated as the ratio of annual precipitation to potential evapotranspiration (http://www.csi.cgiar.org), summer direct normal insolation (“solar radiation” hereafter, http://www.nrel.gov/gis/data_solar.html), elevation (approximated using the digital elevation model gtopo30, https://lta.cr.usgs.gov/GTOPO30), soil moisture (summer average calculated from the daily June–August data from the European Space Agency database, http://www.esa-soilmoisture-cci.org/), and the USDA soil order (“soil type” hereafter, http://websoilsurvey.sc.egov.usda.gov/App/HomePage.htm). We decided to include summer solar radiation over the annual solar radiation as we believe that the former better represents a limiting factor for the growth and survival of trees. Both predictors were, however, moderately correlated (*r *= 0.65). To explore potential impacts of dispersal lags due to Pleistocene glaciation, we also include the historical variable “Ice,” identifying locations under the ice sheets during the Last Glacial Maximum (ca. 24,000–18,000 years BP).

### 2.4 Data analysis

#### 2.4.1 Species’ environmental tolerances

We first established links between functional traits and species’ environmental tolerances using two approaches: (i) a literature review and (ii) pairwise Spearman's correlations between functional traits and species’ environmental tolerances within our data. Second, we quantified associations between trait means and variances at each site and environmental predictors.

#### 2.4.2 Assessing environmental filters

Associations between trait means and variances and environmental predictors were quantified using random forests (Breiman, 2001), each based on 1,000 regression trees. Random forest modeling is a powerful machine‐learning technique that combines the predictions of multiple independent regression trees into a robust composite model. We chose this method over parametric general linear modeling methods because relationships between environmental variables and trait variation are complex at broad scales, often being nonlinear. Random forest modeling is able to disentangle interacting effects and identify nonlinear relationships that often occur at the scale of the analysis performed here among multiple correlated predictors (Cutler et al., 2007). A total of 34 models were generated using the R package *randomForest* (Liaw & Wiener, 2002). For each model, we recorded the percentage of the explained variance (pseudo‐*R* ^2^s) and also ranked the importance of each predictor from 100 (the strongest predictor) to 0 (no predictive power) according to the node purity values (Breiman, 2001). Hence, our models identify traits with strong spatial structure defined by the physical environment (high *R* ^2^s) versus those with noisy or no spatial patterns in either mean values or variances across sites. We assessed the sign of the general relationship between trait means and variances and environmental variables with Pearson's correlations. Additionally, we carried out partial dependence curves for all trait–environment combinations to provide a better understanding of the sign of these relationships. The overall statistical importance of the environmental predictors to the suite of tree traits at our disposal was evaluated by comparing the distributions of importance values from the full set of random forest models.

**Figure 1:**
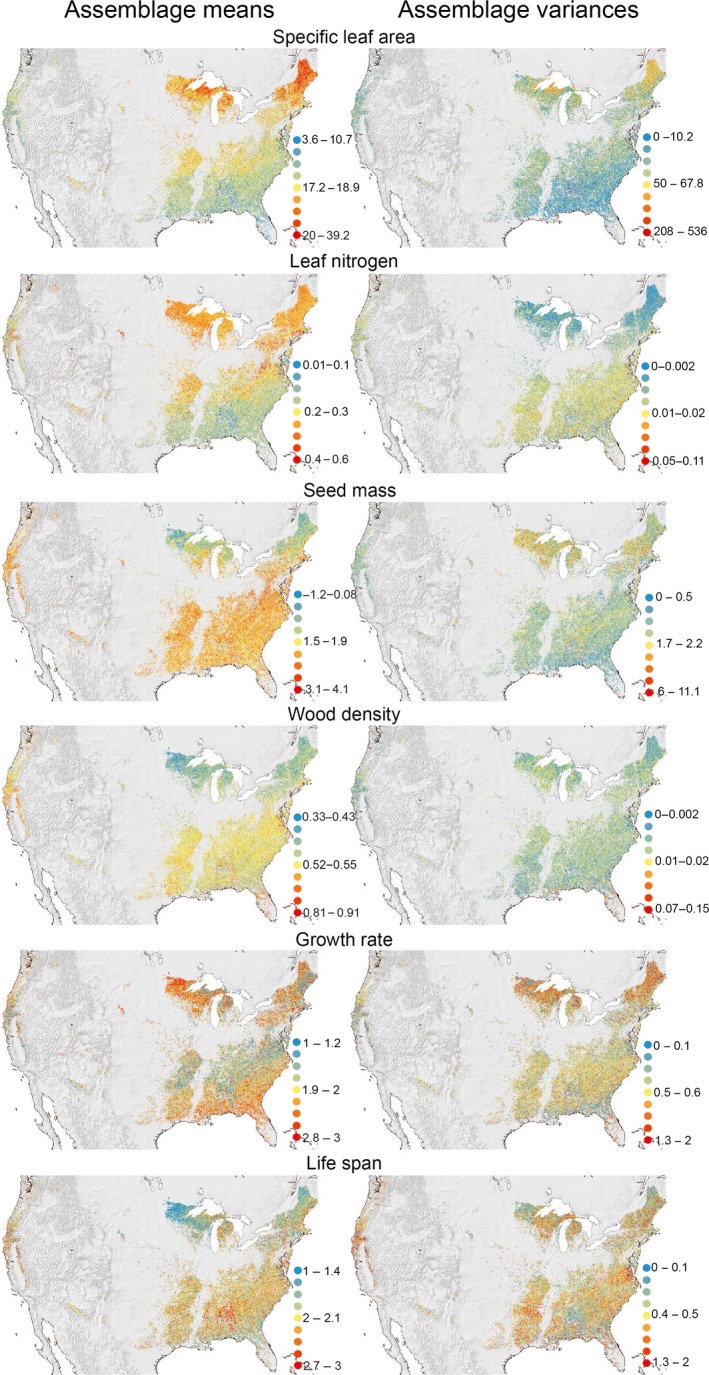
Maps of assemblage means (left column) and variances (right column) for those functional traits with the strongest spatial structure according to the *R* ^2^ of the random forest models for assemblage means (Table 3). See Table 1 for units and Figs S3‐S5 in AppendixS4 for all 34 trait maps

Next, all trait means and variances were mapped into geographical space, as visual inspection of spatial patterns can facilitate the generation of hypotheses of potential drivers, and maps are often easier to interpret than the results of complex analytical algorithms. Due to space constraints, we show here only the maps of those functional traits with the strongest relationship with the environment (Figure 1). In any case, all the 34 maps are provided as supplemental files (Figs S3–S6 in Appendix S4).

Calculating means or variances in the trait values of species assemblages can, however, lead to potential inflations of the coefficient of determination in trait–environment relationships due to the generation of potentially spurious spatial structure and autocorrelation, a problem that has been shown to exist in the FIA data (Hawkins et al., 2017). This is because assemblage trait means and variances mirror the variation in species composition that strongly follows environmental gradients (Zelený & Schaffers, 2012). Attempts to generate appropriate null approaches to resolve this issue have not yet been successful (David Zelený, pers. comm.), leaving the problem of overfit of models analytically unresolvable for the time being. This also greatly complicates meaningful evaluation of the levels of spatial autocorrelation in geographic data and its control, as the standard statistical methods used to control autocorrelation do not resolve the problem (Hawkins et al., 2017). A potential solution is to generate independent statistical evidence for the relationships between traits and environment, so we performed multiple regression analyses at the species level for each trait, which contain no spatial autocorrelation or species co‐occurrences in the response variable. Our reasoning is that if the assemblage‐level and species‐level approaches generate substantially different results, interpretation of the former may not be robust. We selected multiple regression over random forest models because the low number of replicates increases the inter‐tree correlation and therefore can inflate the error rate of the random forest model (Breiman, 2001). Specifically, we attempted to explain the species‐level trait values using the environmental characteristics of the species distributional range (similar approach as in Stahl, Reu, & Wirth, 2014). We generated the environmental variables by calculating the average, minimum, and maximum value of each variable across all sites where each species occurs. Environmental predictors were the same as in the random forest models, but the categorical variables soil type and Ice were excluded. The results based on species‐level traits in multiple regressions supplement the results based on assemblage trait means used in the random forest analyses.

## 3 RESULTS

### 3.1 Species’ environmental tolerances

Our predictions with respect to the major stressors based on the literature largely corresponded to the results obtained from the correlations between functional traits and environmental tolerances at the species level (Table 2, see also Table S2 in Appendix S5). Even so, there were some notable differences. First, specific leaf area and leaf nitrogen were more strongly related to cold tolerance than to drought or shade tolerance, although the correlations with drought tolerance were relatively strong as well (Table S2). And, instead of the expected relationship with drought tolerance, tree height was better, although weakly, associated with cold tolerance. Second, bark thickness best corresponded to the shade tolerance rather than fire tolerance. Third, instead of the expected relationship with drought or shade tolerance, tree lifespan best correlated with fire tolerance.

**Table 2:** The functional traits, their strongest environmental tolerance correlated with the sign of the correlation coefficient, and the expected environmental filter(s) to which each trait is presumed to respond based on the literature

Trait	Best species‐level correlate (*r*)	Expected filters
SLA	Cold tolerance (−0.48)	Drought/shade/nutrients
Leaf N	Cold tolerance (−0.32)	Drought/shade/nutrients
Leaf P	Shade tolerance (−0.15)	Drought/shade/nutrients
Leaf shape	Waterlogging tolerance (−0.08)	Drought/waterlogging
Seed mass	Drought tolerance (0.29)	Coldness/drought/shade
Dispersal mode	Cold tolerance (−0.33)	Coldness/drought
Wood density	Drought tolerance (0.51)	Drought/waterlogging
Growth rate	Shade tolerance (−0.26)	Drought/shade/nutrients
Lifespan	Fire tolerance (−0.25)	Drought/shade/nutrients
Height	Cold tolerance (−0.16)	Drought/waterlogging
Bark thickness	Shade tolerance (−0.24)	Fire
Winter buds size	Drought tolerance (−0.16)	Drought/shade

See Appendix S5 for the full correlation matrix.

### 3.2 Assessing environmental filters of assemblage trait means

Minimum temperature was the dominant predictor of the spatial variation in the assemblage‐level means of the majority of functional traits, and the percentage of variance explained in this subset of models was the highest (Table 3). Aridity index, evapotranspiration, solar radiation, summer precipitation, maximum temperature, and elevation also explained some of the spatial structure in trait means, whereas occurrence of historic glaciation, soil moisture, and soil types was poor predictors.

**Table 3:** Random forest models (1,000 regression trees) for mean trait values across 74,689 FIA sites, grouped by the most important predictor variable and ranked by the explanatory power (percentage of variance explained) of the model

	*R* ^2^	Min T	Max T	Sum P	ET	Arid Index	Sol rad	Ice	Soil moist	Elev	Soil type
Cold tolerance	0.83	**100 (+)**	55	31	8	13	24	51	10	28	41
Leaf N	0.61	**100 (−)**	43	39	23	31	41	18	21	46	55
SLA	0.60	**91 (−)**	**100 (−)**	40	31	57	41	44	25	49	50
Drought tolerance	0.59	87	**100 (+)**	43	32	48	60	46	28	43	66
Seed mass	0.58	**100 (+)**	77	27	28	36	36	42	24	32	49
Dispersal mode	0.54	**100 (−)**	73	30	32	39	39	63	25	34	46
Fire tolerance	0.54	**100 (−)**	58	51	34	53	52	33	28	60	38
Wood density	0.48	**100 (+)**	64	47	41	54	64	43	32	41	42
Waterlogging tolerance	0.43	**92 (+)**	54	76	51	73	78	9	38	**100 (−)**	42
Growth rate	0.32	84	51	72	66	**100 (−)**	88	6	47	76	44
Shade tolerance	0.30	**91 (−)**	60	57	69	**100 (+)**	84	5	51	67	36
Life span	0.30	**100 (+)**	58	61	64	**90 (+)**	79	12	46	57	45
Height	0.28	**100 (+)**	74	60	69	79	83	39	47	67	36
Leaf P	0.26	89	61	70	77	**100 (−)**	**95 (+)**	9	58	71	56
Leaf shape	0.22	75	85	59	80	**100 (+)**	86	9	51	69	57
Bark thickness	0.19	**93 (+)**	75	61	83	**100 (−)**	89	14	57	71	32
Winter buds size	0.14	69	58	66	85	**100 (+)**	87	2	54	70	21

The sign of the Pearson correlation of the trait and environmental variable is represented by ± beside the most important predictor. “Min T” = minimum winter temperature, “Max T” = maximum summer temperature, “Sum P” = summer precipitation, “ET” = evapotranspiration, “Arid Index” = aridity index (the ratio of annual precipitation to potential evapotranspiration), “Ice” indicates whether the area was glaciated or not during the Last Glacial Maximum, “Soil moist” = soil moisture, “Sol rad” = solar summer radiation, “Elev” = elevation. See Appendix S7 for the partial dependence curves.

Minimum temperature was the best predictor of most of the functional traits associated with cold and drought tolerance (Table 2). These included (i) leaf economic spectrum traits (Wright et al., 2004) such as leaf N and SLA, where assemblage means decreased with increasing temperature (Table 3; Figure 1), although for SLA maximum temperature performed slightly better, (ii) seed dispersal traits such as seed size and seed dispersal mode, and (iii) tree lifespan and traits related to stem hydraulics such as wood density and leaf maximum tree height, for which assemblage means increased with increasing temperature (Table 3, Figs 1 and S3–S4 in Appendix S4).

Aridity index best explained the means of those functional traits related to shade tolerance (growth rate, bark thickness, and leaf P), where assemblage means increased with increasing aridity (i.e., decreased with increasing aridity index defined as the ratio of annual precipitation to potential evapotranspiration (Table 3, Figs 1 and S3–S4 in Appendix S4). Aridity index further explained variation in the means of winter bud size and leaf shape.

With some exceptions, these results were supported by the multiple regressions at the species level (Appendix S6). Minimum temperature was the strongest predictor for species‐level leaf N and SLA. Temperature remained the best predictor of wood density, fire tolerance, lifespan, and drought tolerance, although these traits better corresponded to the maximum instead of the minimum temperature. Although species‐level height was best explained by soil moisture, the effect of minimum temperature was relatively strong as well. Also, variables related to water availability (summer precipitation, evapotranspiration, and soil moisture) remained the best predictors of species‐level leaf P. Nevertheless, instead of minimum temperature, species‐level seed mass was best explained by solar radiation. And lastly, instead of elevation, species‐level waterlogging tolerance was best explained by maximum temperature, solar radiation, and soil moisture. For the rest of the traits (bark thickness, growth rate, leaf shape, winter bud length, dispersal mode), multiple regression models were weak.

### 3.3 Assessing environmental filters of assemblage trait variances

Patterns of trait variances were substantially weaker than those for means in all cases. *R* ^2^s of the random forests for means averaged 0.36 for functional traits only, whereas for the modeled variances, *R* ^2^s averaged 0.17, suggesting that levels of stochasticity in variances are substantially higher than for trait means. Still, most patterns of trait variances had some level of spatial structure associated with environmental conditions (Table 4; Figure 1). Similar to assemblage means, aridity index best explained assemblage variances in traits related to shade tolerance (bark thickness, growth rate, and leaf P) and variances in leaf shape, and winter buds size. Still, the variation explained by these models was low, ranging from 0.06 to 0.18.

**Table 4:** Random forest models (1,000 regression trees) for trait variances across 74,689 FIA sites, grouped by the most important predictor variable and ranked by the explanatory power (percentage of variance explained) of the model

	*R* ^2^	Min T	Max T	Sum P	ET	Arid Index	Sol rad	Ice	Soil moist	Elev	Soil type
Cold tolerance	0.45	**94 (+)**	46	**100 (+)**	41	62	52	6	31	71	28
Leaf N	0.32	**100 (+)**	62	46	53	62	59	47	38	51	37
Drought tolerance	0.28	**100 (+)**	73	44	58	68	58	32	36	53	39
Height	0.27	**97 (+)**	**100 (+)**	74	82	**97 (−)**	**90 (−)**	32	54	69	77
Seed mass	0.23	81	77	62	77	**100 (−)**	84	13	51	70	30
Fire tolerance	0.22	**100 (−)**	70	64	78	88	79	13	48	65	36
SLA	0.22	78	78	62	76	**100 (+)**	83	15	47	66	36
Wood density	0.18	84	60	73	75	**100 (−)**	77	2	77	64	26
Bark thickness	0.18	74	64	60	74	**100 (−)**	83	3	48	64	31
Leaf shape	0.17	74	78	61	80	**100 (+)**	89	6	56	72	25
Dispersal mode	0.17	79	71	68	85	**95 (+)**	**100 (−)**	10	56	77	35
Shade tolerance	0.16	80	76	59	87	**100 (+)**	88	16	51	68	37
Winter buds size	0.11	66	55	61	**91 (−)**	**100 (−)**	**92 (+)**	2	53	66	18
Waterlogging tolerance	0.09	72	66	67	**93 (−)**	**100 (+)**	**90 (−)**	17	52	72	30
Growth rate	0.06	71	56	65	**90 (−)**	**100 (+)**	87	3	53	69	18
Life span	0.09	67	59	70	**90 (+)**	**100 (−)**	**92 (+)**	2	54	71	19
Leaf P	0.06	81	58	67	**92 (+)**	**100 (+)**	89	11	54	69	24

The sign of the Pearson correlation of the trait and environmental variable is represented by ± beside the most important predictor. See Table 3 for explanation of abbreviations of environmental variables.

The expectation that minimum temperature would constrain both means and variances of the same functional traits was not confirmed (with the exception of leaf N). Instead, aridity index became the dominant predictors of the assemblage variances of those traits related to cold and drought tolerance. The direction and form of these relationships, however, varied among traits (Table 4; see also partial dependence curves in Appendix S7).

## 4 DISCUSSION

We found that coldness and drought are the most important environmental stressors constraining forest assemblage trait means across the conterminous USA. Most of our predictions were fulfilled, although relationships between particular traits and environmental variables vary somewhat depending on whether a community‐ or species‐level approach is used. We found that minimum temperature best predicted community trait means of leaf economic spectrum traits (leaf N and SLA) and dispersal traits (seed mass and mode of dispersion). It also best predicted assemblage trait means of traits related to stem hydraulics (wood density and height). Most of these traits were related to cold tolerance, but unexpectedly this group also contains traits that have been related to drought tolerance (seed mass and wood density). We further found that aridity index best explained community means of traits related to shade tolerance (growth rate, leaf P, and bark thickness). Nevertheless, the explanatory power of this subset of models was not as strong, probably because in contrast to temperature, gradients of precipitation or solar radiation have a more regional pattern at continental scales. Our results thus confirm previous studies considering tree tolerance to coldness as the most important adaptation constraining the large‐scale distribution of woody plants (Hawkins et al., 2014; Šímová et al., 2011). Stress from shade represents, on the other hand, another constrain acting independently on the temperature gradient, although with much weaker effect at the continental scale.

**Figure 2:**
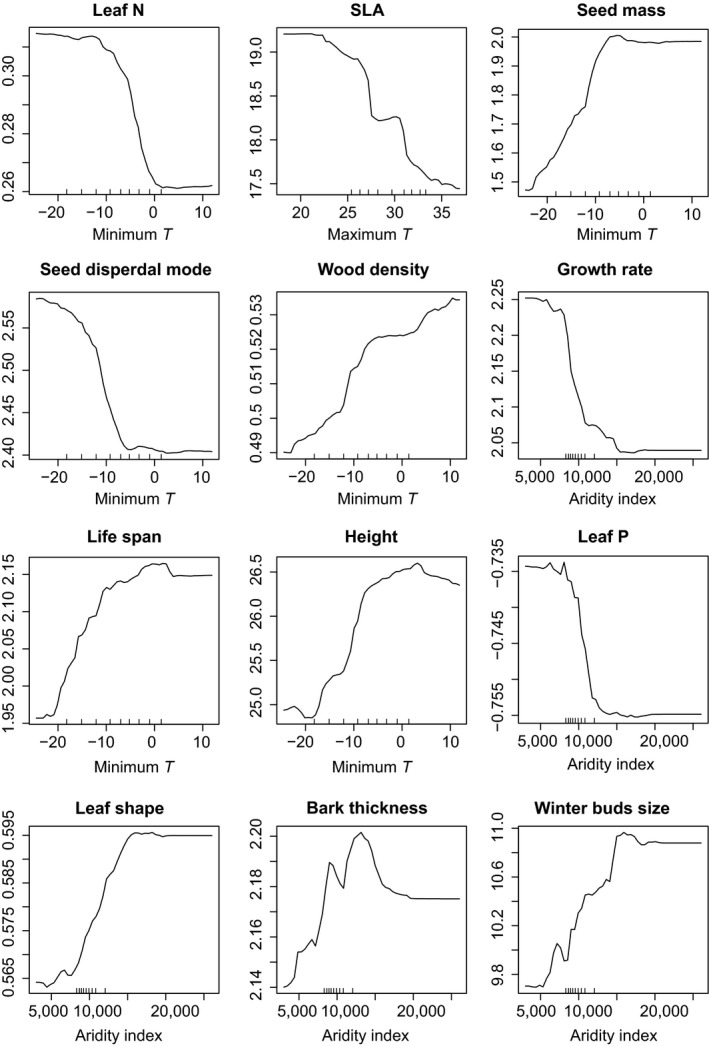
Partial dependence plots (response curves) for the 12 functional trait means and their best environmental predictors identified by the random forest analysis (Table 3)

More specifically, and as we expected, assemblage means of dispersal traits (seed mass and dispersal mode) were best explained by minimum temperature. Decreasing mean seed mass and an associated shift in dispersal mode toward the north is consistent with wind being the main dispersal factor for seeds in colder environments, whereas warm conditions favor animal dispersal (Howe & Smallwood, 1982). Interestingly, and in contrast to our prediction, minimum temperature constrained community means of tree height and wood density, that is, those traits associated with stem hydraulics and the plant capacity to transport water. At a first glance, these results contradict the hypothesis that short trees with dense wood can better resist the embolism caused by the drought stress (Hacke, Sperry, Pockman, Davis, & McCulloh, 2001; Ryan & Yoder, 1997). However, although mean wood density increased with minimum temperature relatively steeply (Figure 2), the relationship between minimum temperature and mean assemblage height was unimodal—tall trees occurred mostly around 0°C (Figure 2). This indicates that both high and low values of temperature act as stressors for tree size, whereas relatively mild environments might facilitate dense forests with trees competing for light, leading to the high assemblage means of these two traits (Moles et al., 2009).

Although we expected specific leaf area (SLA), leaf nutrient traits (leaf N and leaf P), and whole plant growth and mortality rate (lifespan) to follow similar trade‐off axes of drought, nutrient, or shade stress, their response to environmental variables as well as to species’ environmental tolerances differed. SLA and leaf N were primarily associated with cold tolerance and their assemblage means increased with maximum and minimum temperature, respectively. The increase in the proportion of angiosperm trees having high SLA and leaf N content per mass toward lower temperatures is consistent with previous findings (Cates & Orians, 1975; Royer, Peppe, Wheeler, & Niinemets, 2012; Wright et al., 2004). It is also in line with the hypothesis that trees from cold sites require high photosynthetic rates to adapt to a short growing season, whereas nutrient‐limited subtropical sites from low latitudes favor evergreen species investing into leaf structure and associated nutrient conservation (Chabot & Hicks, 1982; Ordoñez et al., 2009). Besides the direct stress from cold or freezing, low temperature likely limits species distribution through its negative effect on the growing season length (Morin, Augspurger, & Chuine, 2007). In contrast to leaf N and SLA, increasing leaf P and growth rate best corresponded to decreasing shade tolerance and their assemblage means were best explained by increasing aridity. This result agrees with the evidence that unlike fast growing light‐demanding species, shade‐tolerant species survive with lower growth rate (Kitajima, 1994; Reich et al., 2003). In contrast to our expectation, another trait negatively correlated with shade tolerance was bark thickness. This finding can be interpreted by the fact that some shade‐tolerant trees can persist in the forest understory until a gap opening promotes their accelerated growth (Denslow, 1980), that may not be possible with thick bark.

It is intriguing that minimum temperature was associated with assemblage means for leaf economic spectrum and dispersal traits, whereas their variances, with variable patterns, were associated with aridity. Although these relationships were rather weak and variable in their directions, water availability seems to have a major impact on community functional diversity. These results, despite a few exceptions, do not support our expectation that trait variances should be limited by the same stressors that constrains the mean of a particular trait in an assemblage. On the other hand, the observed weak evidence of the environment reducing trait variances is consistent with recent findings (Coyle et al., 2014; Šímová et al., 2015). One possible explanation is that trait variances are partially constrained by competition, which is typically more intense in benign climates (Enquist et al., 2015; Godoy, Kraft, & Levine, 2014). Whatever the cause, our reasoning is limited by the fact that we cannot provide independent statistical evidence (such as the species‐level correlations) to support the results concerning trait variances, and hence, we cannot reject the hypothesis that such patterns emerged stochastically.

Our results could be affected by five potential biases. First, we did not consider the influence of biotic interactions. Environmental filtering is generally defined as “abiotic factors that prevent the establishment or persistence of species in a particular location” (Kraft et al., 2015). Hence, without accounting for other factors that may shape the community functional composition such as biotic interactions (e.g., competition, herbivory, host‐pathogen coevolution) or dispersal limitation, we may be overstating the role of environment. However, and despite their potential importance, to date it is impossible to obtain such data at large spatial scales. Second, we could have missed important environmental factors that could have been captured by including geographical space (e.g., latitude or longitude) as predictor variables (Pavoine, Vela, Gachet, de Bélair, & Bonsall, 2011). But, there is a strong correlation between geographical and environmental variables in our data; in particular, latitude is strongly correlated with maximum (*r *= −0.86) and minimum temperature (*r *= −0.78). Correlations of biological patterns with spatial variables are also difficult to interpret ecologically given the complex covariance structures among locations and both measured and unmeasured environmental gradients at broad extents (Hawkins & Diniz‐Filho, 2004). Third, the species composition could be altered by the legacies from the past. Although we focused our analyses on natural forest stands, these sites were likely managed and some of them could still be managed to some extent. Unfortunately, the separation of human impact from nature is nearly impossible. Fourth, we have handled particular species as independent units, ignoring their evolutionary history. Therefore, including phylogenetic information would represent an interesting next step (de Bello et al., 2015; Prinzing, 2016; Šímová, 2016). Fourth, we used a single species‐level value for each trait, ignoring local adaptation. Unfortunately, intraspecific measurements across the entire USA do not exist for the vast majority of traits or species, so there is no recourse, except to note that our analysis is missing potentially important gradients operating at the population level. This is virtually always the case in broad‐scale analyses of tree traits. The expectation is that incorporating intraspecific variation would increase the strength of spatial gradients we found, but we have no evidence that this is true.

To conclude, using multiple traits and environmental predictors, we found that assemblage means of traits associated with species abilities to cope with both coldness and drought vary along temperature axis. Temperature is thus the most important driver of the functional community structure of North American forests. In turn, albeit more weakly, water availability affects those traits related to shade tolerance, much likely through their effects on the forest canopy structure and vegetation openness. Understanding the links between environment, species’ environmental tolerances and species functional traits is key for dynamic global vegetation models (DGVMs) predicting species distribution under the global change. Unfortunately, vegetation properties in these models are represented by separate vegetation units rather than continuous values of plant functional traits. Therefore, our results have important implications for the much currently needed task of incorporating plant functional traits into DGVMs.

## CONFLICT OF INTEREST

None declared.

## AUTHOR CONTRIBUTION

BAH, IS, and MR designed the study, MR analyzed the data, IS led the writing with the major contribution of MR and BAH.
