# Supplementary material for: Stress from cold and drought as drivers of functional trait spectra in North American angiosperm tree assemblages
Source: Ecol Evol. 2017 Aug 14;7(18):7548–59. doi: 10.1002/ece3.3297 (PMC5606901; doi:10.1002/ece3.3297)
Supplement: Supplementary file 1 [file ECE3-7-7548-s001.docx]

SUPPORTING INFORMATION

Stress from cold and drought as drivers of functional trait spectra in North American angiosperm tree assemblages

Irena Šímová, Marta Rueda, Bradford A. Hawkins

Appendix S1 – Explanation of species‘ environmental tolerances.

**Minimum temperature**

Source: USDA database (plants.usda.gov)

Description: The minimum tolerable temperature is the lowest temperature recorded in the plant’s historical range. If this is not available, the record low January temperature recorded at climate stations within the current geographical range of the plant is used.

Units: °F

**Fire Tolerance**

Source: USDA database (plants.usda.gov)

Description: What is the relative ability to resprout, regrow, or reestablish from residual seed after a fire?

Units: Ordinal (1-4): 1, None; 2, Low; 3, Medium; 4, High.

**Shade tolerance**

Source: Niinemets & Valladares (2006)

Description: The capacity for growth in the shade.

Units: Cardinal (1-5): 1,very intolerant; 2, intolerant; 3, moderately tolerant; 4,tolerant; 5, very tolerant. This scale corresponds approximately to the following light availabilities expressed as percentage of full sunlight: 1, 50%; 2, 25–50%; 3, 10–25%;4,5–10%; 5, 2–5%.

**Waterlogging tolerance**

Source: Niinemets & Valladares (2006)

Description: Tolerance of reduced root-zone soil oxygen availabilities.

Units: Cardinal (1-5): 5, very tolerant (survives deep, prolonged waterlogging for more than one year); 4, tolerant (survives deep water-logging for one growing season); 3, moderately tolerant (survives waterlogging or saturated soils for 30 consecutive days during the growing season); 2, intolerant (tolerates one to two weeks of waterlogging during the growing season); 1, very intolerant (does not tolerate water-saturated soils for more than a few days during the growing season).

**Drought tolerance**

Source: Niinemets & Valladares (2006)

Description: Physiological tolerance to water stress, morphological and life cycle strategies to cope with scant water, and the water availability estimated on the sites where the species more frequently occur.

Units: Cardinal (1-5): 1, very intolerant; 2, intolerant; 3, moderately tolerant; 4, tolerant; 5, very tolerant

1, 600 mm precipitation with little variation during growing season, P:PET ratio of 3.0, few days of drought, and greater than 0.3 MPa soil water potential; 2, 500–600 mm precipitation, variation of precipitation distribution during growing season characterized by coefficient of variation,10%, P:PET ratio of 1.5:3, few weeks of drought, and from 0.3 to 0.8 MPa soil water potential; 3, 400–500 mm precipitation with a growing season coefficient of variation of 10–15%,P:PET ratio of 0.8:1.5, up to one month of drought, and from 0.8 to 1.5 MPa soil water potential; 4, 300–400 mm precipitation with a growing season coefficient of variation of 20–25%, P:PET ratio of 0.5:0.8, two to three months of drought, and from 1.5 to 3 MPa soil water potential; 5, 300 mm precipitation with a growing season coefficient of variation. 25%, P:PET ratio of 0.5, more than three months of drought, and less than 3 MPa soil water potential.

**Literature**

Niinemets, Ü., & Valladares, F. 2006. Tolerance to shade, drought, and waterlogging of temperate Northern Hemisphere trees and shrubs. *Ecological Monographs* 76: 521–547.
