# Supplementary material for: Stress from cold and drought as drivers of functional trait spectra in North American angiosperm tree assemblages
Source: Ecol Evol. 2017 Aug 14;7(18):7548–59. doi: 10.1002/ece3.3297 (PMC5606901; doi:10.1002/ece3.3297)
Supplement: Supplementary file 3 [file ECE3-7-7548-s003.docx]

SUPPORTING INFORMATION

Stress from cold and drought as drivers of functional trait spectra in North American angiosperm tree assemblages

Irena Šímová, Marta Rueda, Bradford A. Hawkins

Appendix S3 – Correlations among assemblage means/variances of different traits.


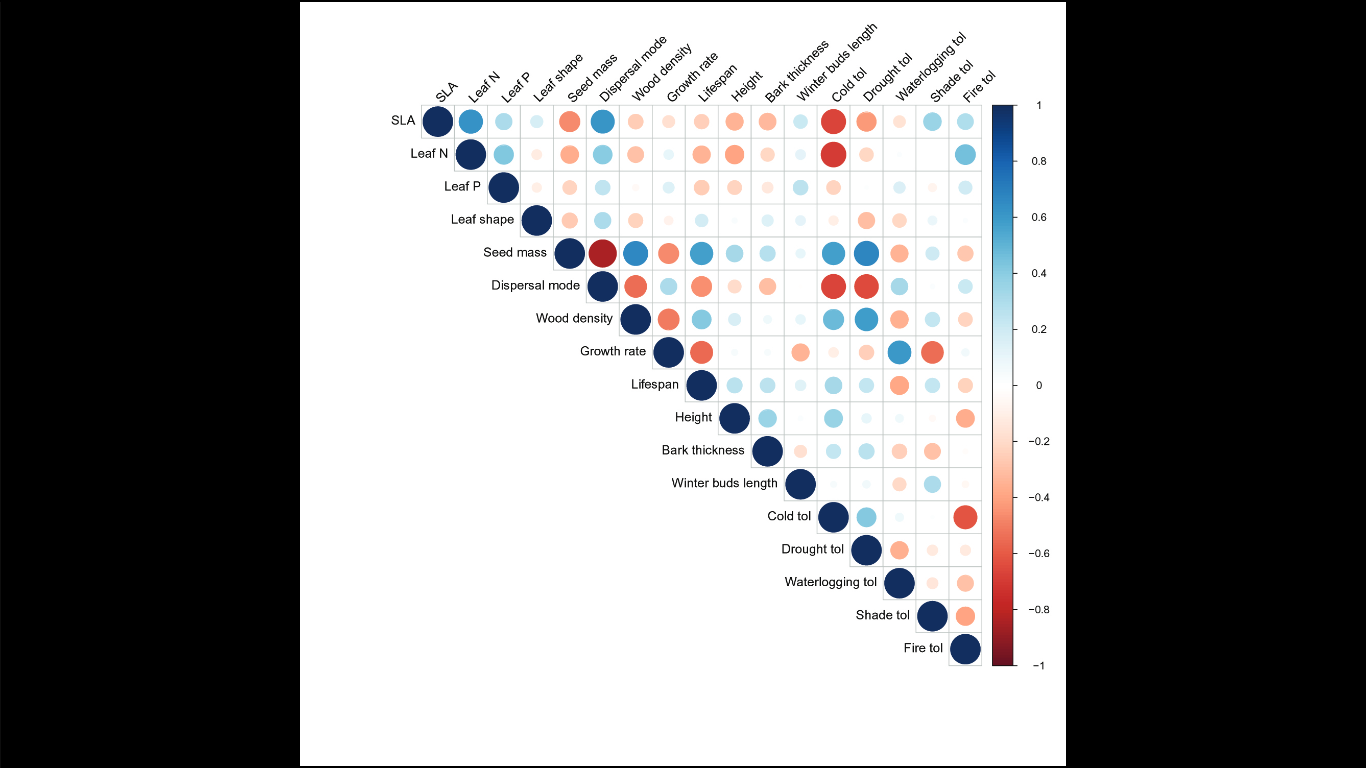


Figure S1: Pearson correlation coefficients among assemblage means of different traits.


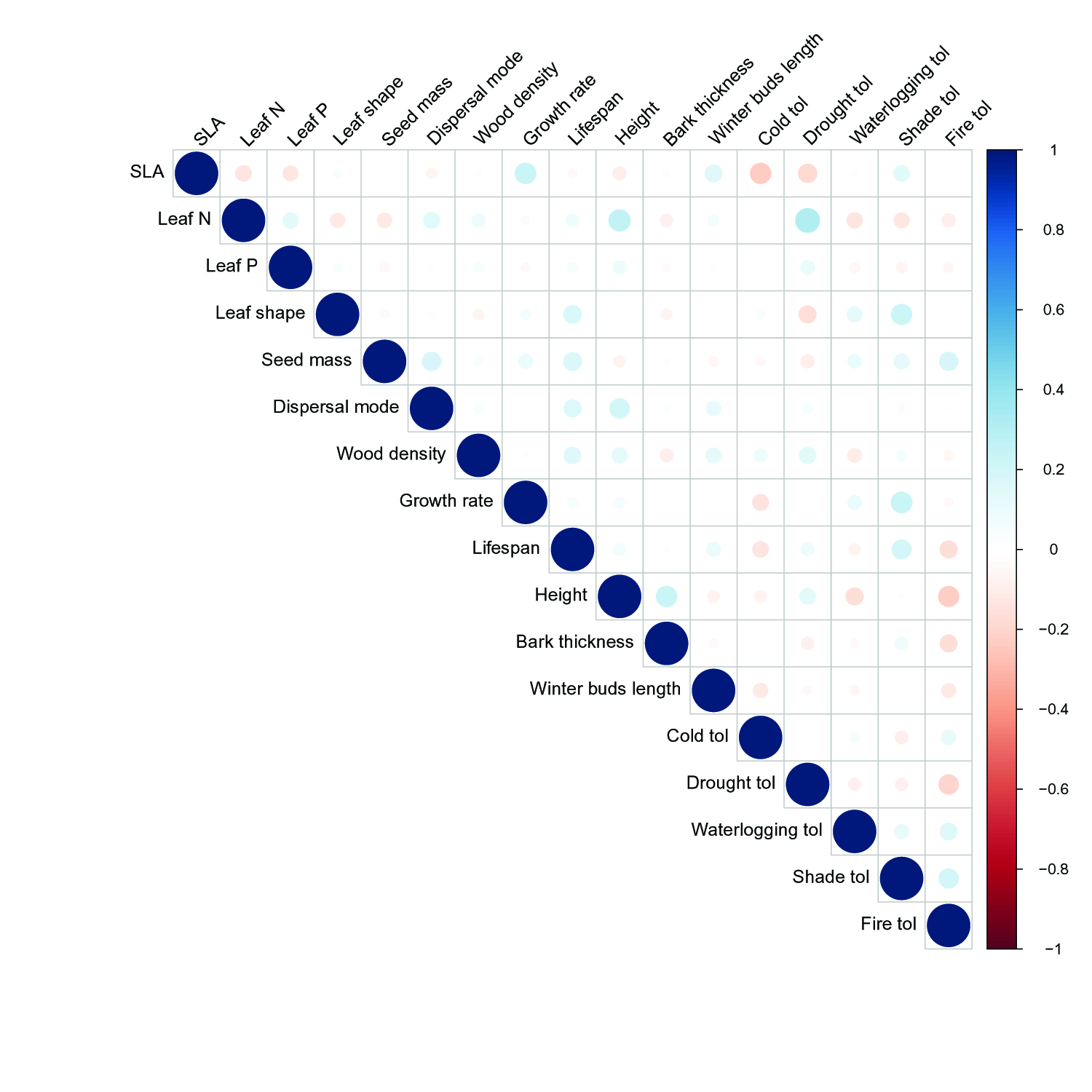


Figure S2: Pearson correlation coefficients among assemblage means of different traits.

Appendix S4 - Maps of assemblage trait means and variances.


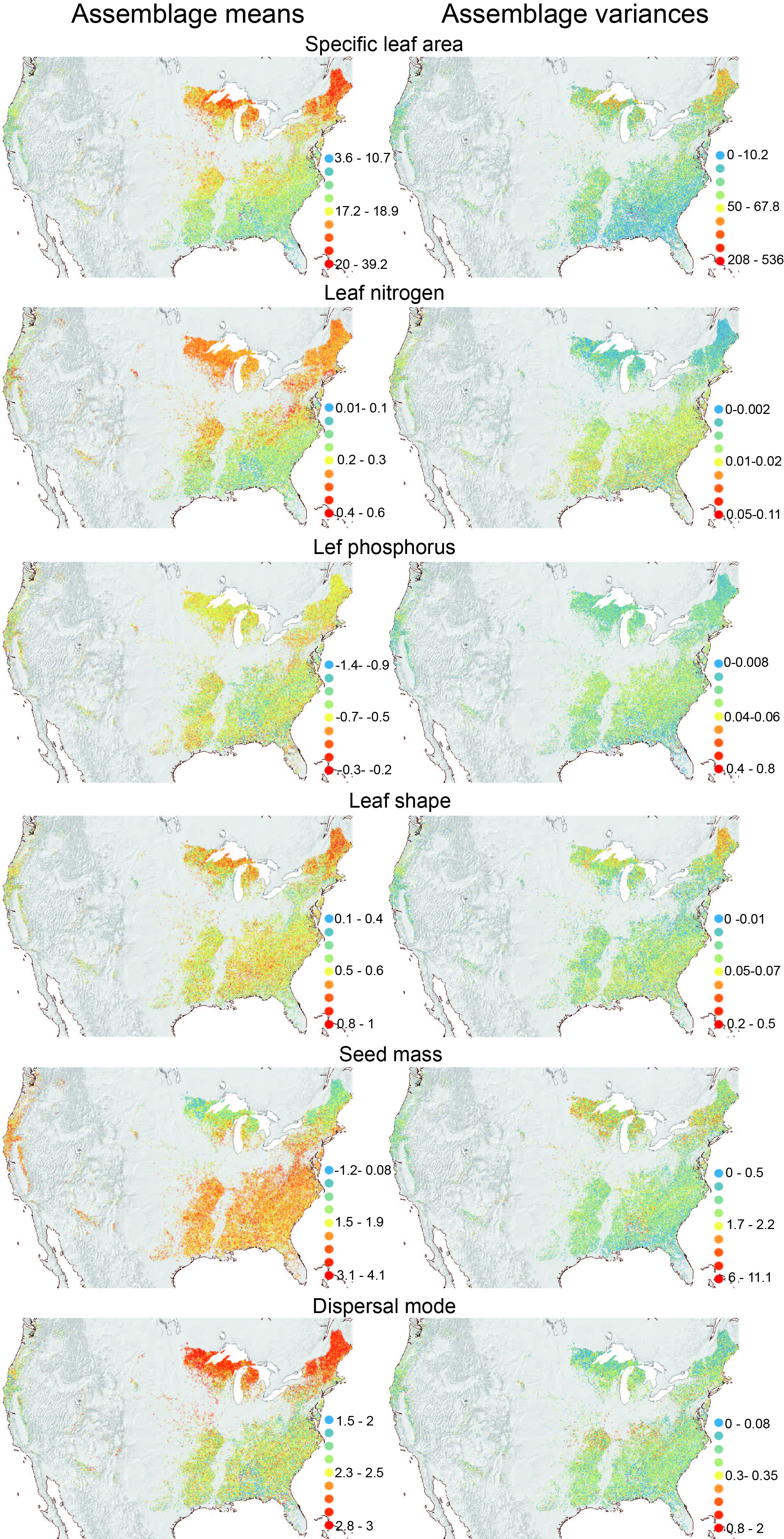


Figure S3: Maps of assemblage means (left column) and variances (right column) of leaf and dispersal traits.


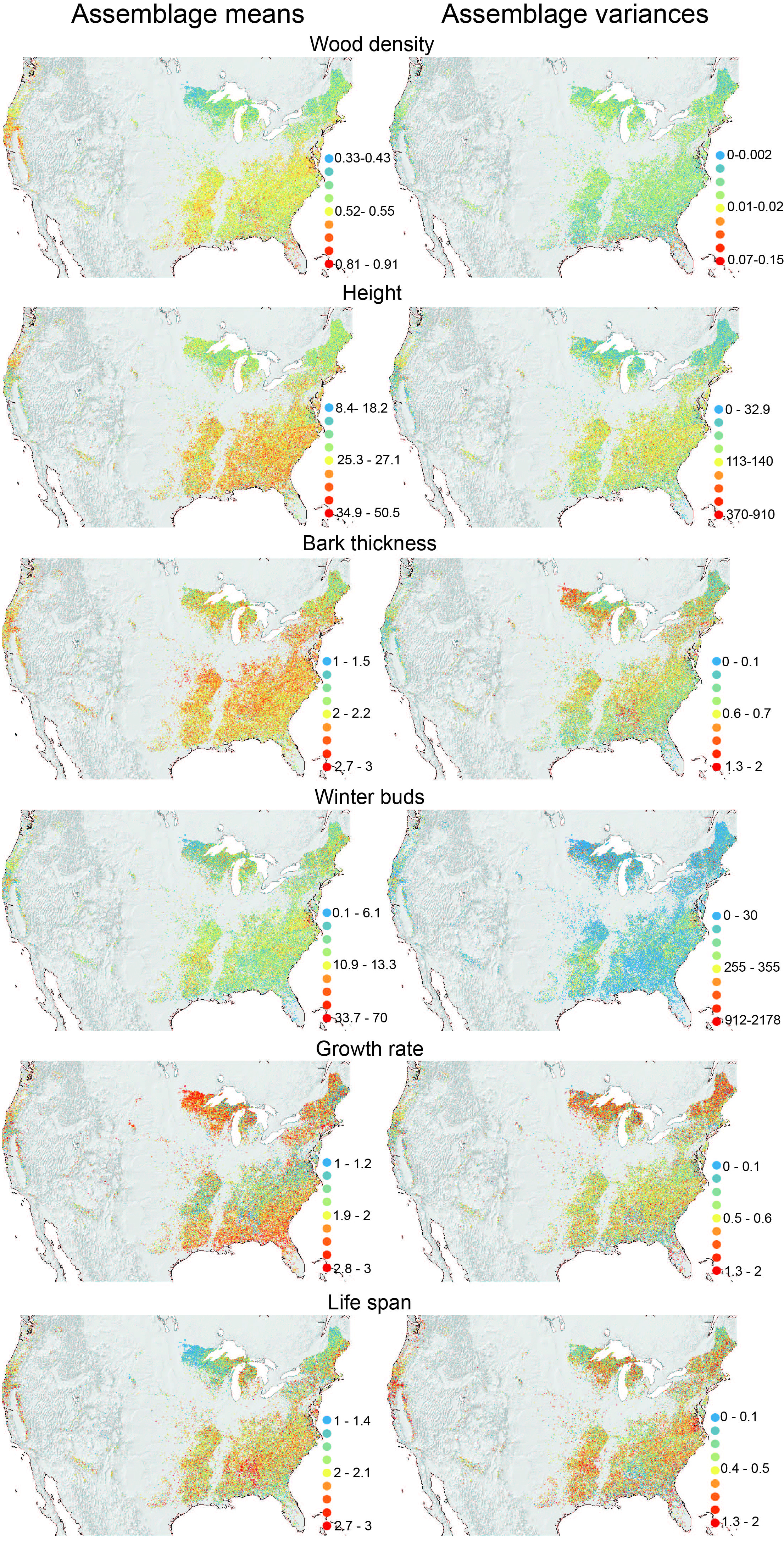


Figure S4: Maps of assemblage means (left column) and variances (right column) of traits related to tissue structure and tree lifespan.


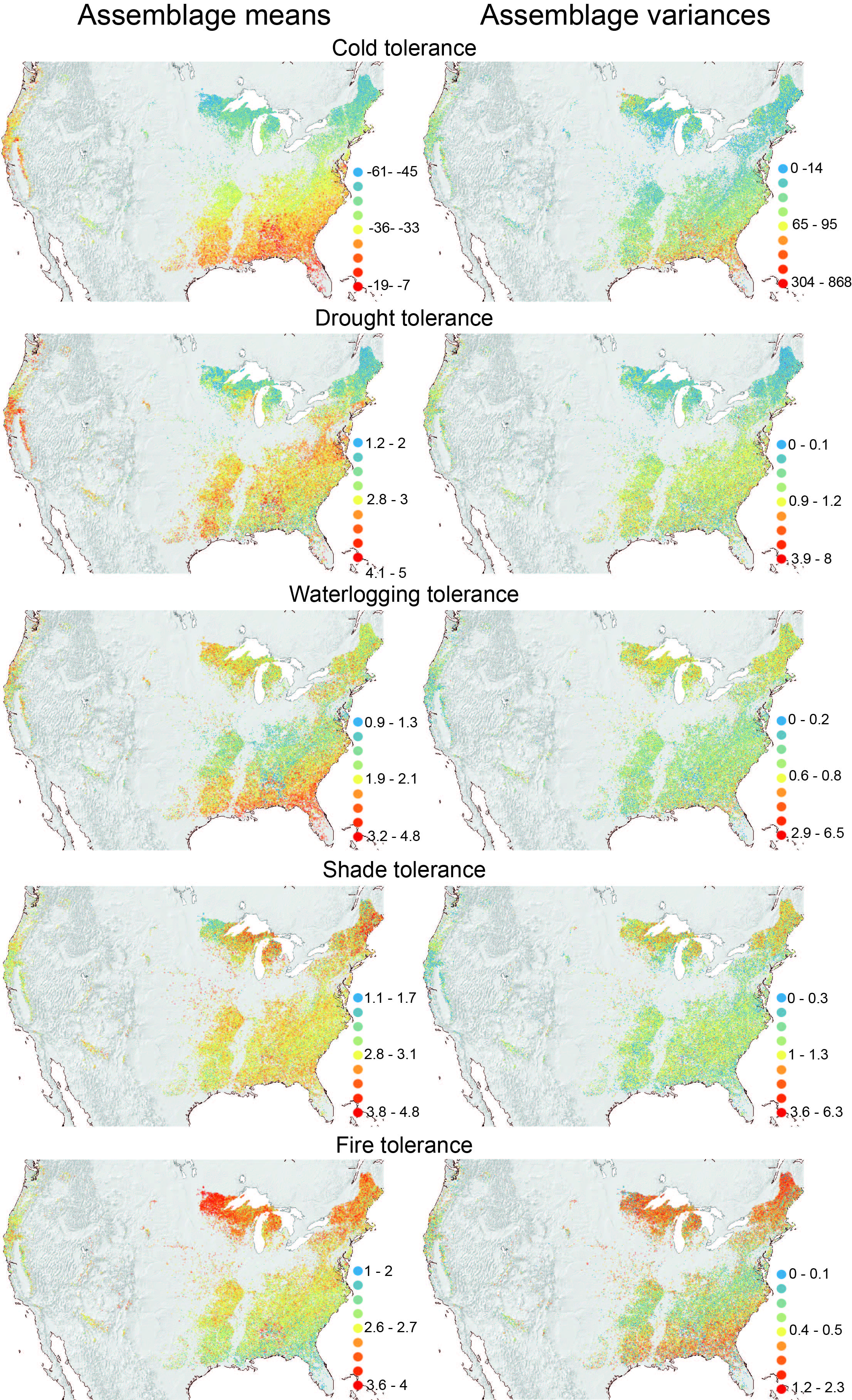


Figure S5: Maps of assemblage means (left column) and variances (right column) of species‘ environmental tolerances.

Appendix S5 – Relationships between traits and species‘ environmental tolerances

Table S2: Pairwise Spearman correlation coefficients between performance and functional traits. The strongest correlations for each functional trait is highlighted in bold.

|  | Seed mass | Dispersal mode | Height | Leaf N | Leaf P | SLA | Leaf shape | Growth rate | Life span | Bark thickness | Winter buds size | Wood density |
| --- | --- | --- | --- | --- | --- | --- | --- | --- | --- | --- | --- | --- |
| Waterlogging tol | -0.270 | 0.193 | 0.058 | 0.239 | 0.015 | 0.073 | **-0.083** | 0.172 | -0.136 | -0.076 | -0.094 | -0.270 |
| Drought tol | **0.293** | -0.217 | -0.028 | -0.290 | 0.064 | -0.295 | 0.029 | -0.175 | 0.148 | 0.189 | **-0.162** | **0.513** |
| Fire tol | -0.154 | 0.136 | -0.036 | 0.018 | -0.047 | 0.156 | 0.024 | 0.132 | **-0.247** | -0.063 | -0.095 | 0.007 |
| Shade tol | 0.184 | -0.144 | -0.004 | 0.003 | **-0.148** | 0.231 | 0.000 | **-0.261** | -0.011 | **-0.241** | 0.104 | -0.073 |
| Cold tol | 0.195 | **-0.330** | **-0.164** | **-0.315** | -0.139 | **-0.484** | -0.043 | -0.105 | 0.186 | 0.060 | 0.024 | 0.292 |

Appendix S6 - Results from the multiple regression analyses using species-level traits as response variable.

Table S3: Results from the multiple regression analyses using species-level traits as response variable and *mean values of environmental variables* of the plots where the species occur as explanatory variables. Coefficients bellow each variable are standardized and indicate the relative contribution of this variable within each model. Significant models are highlighted in orange. ‘Min T’ = minimum winter temperature, ‘Max T’ = maximum summer temperature, ‘Sum P’ = summer precipitation, ‘ET’ = evapotranspiration, ‘Arid Index’ = aridity index, ‘Soil moist’ = soil moisture, ‘Solar rad’ = solar summer radiation, ‘Elev’ = elevation.

|  | *R^2^* | *p*-value | Max T | Min T | Sum P | Elev | Soil moist | Solar rad | ET | Arid index |
| --- | --- | --- | --- | --- | --- | --- | --- | --- | --- | --- |
|  |  |  |  |  |  |  |  |  |  |  |
| Cold tolerance | 0.57 | <0.001 | 0.14 | 0.41 | 0.96 | 0.14 | -0.45 | 0.69 | 0.07 | 0.37 |
| Waterlogging tolerance | 0.39 | <0.001 | -0.58 | 0.39 | 0.74 | -0.53 | 0.60 | 0.87 | -0.20 | -0.65 |
| Fire tolerance | 0.26 | <0.001 | -0.80 | 0.26 | -0.20 | -0.33 | 0.05 | -0.10 | -0.16 | -0.37 |
| Leaf N | 0.25 | <0.001 | 0.91 | -1.20 | -0.05 | -0.09 | -0.23 | 0.06 | 0.28 | 0.21 |
| Wood density | 0.24 | <0.001 | 0.63 | -0.06 | 0.32 | 0.20 | -0.46 | -0.10 | -0.27 | 0.12 |
| SLA | 0.24 | 0.002 | 0.24 | -0.54 | -0.19 | 0.10 | 0.02 | -0.17 | 0.14 | 0.24 |
| Leaf P | 0.24 | 0.02 | 0.15 | 0.35 | 1.16 | -0.07 | -0.69 | -0.01 | -0.90 | 0.26 |
| Drought tolerance | 0.19 | <0.001 | 0.73 | -0.44 | 0.30 | 0.45 | -0.48 | 0.13 | 0.04 | 0.22 |
| Life span | 0.15 | 0.001 | 0.61 | -0.16 | 0.09 | 0.46 | -0.04 | 0.22 | -0.07 | 0.28 |
| Shade tolerance | 0.09 | 0.16 | -0.08 | 0.21 | -0.4 | 0.03 | 0.50 | -0.02 | 0.06 | 0.05 |
| Height | 0.08 | 0.08 | 0.10 | -0.13 | 0.15 | -0.11 | 0.21 | 0.46 | -0.07 | 0.18 |
| Dispersal mode | 0.08 | 0.09 | -0.42 | 0.02 | 0.17 | -0.33 | -0.07 | 0.14 | <0.01 | -0.11 |
| Leaf shape | 0.08 | 0.08 | -0.49 | 0.30 | -0.45 | -0.31 | 0.02 | -0.30 | -0.02 | -0.08 |
| Seed mass | 0.07 | 0.18 | 0.33 | -0.16 | 0.01 | 0.06 | -0.24 | 0.12 | -0.01 | 0.26 |
| Growth rate | 0.07 | 0.26 | -0.47 | 0.33 | -0.33 | -0.37 | 0.06 | -0.38 | 0.04 | -0.20 |
| Bark thickness | 0.06 | 0.33 | 0.18 | -0.12 | 0.21 | -0.15 | -0.21 | 0.29 | -0.06 | 0.25 |
| Winter buds size | 0.03 | 0.86 | -0.08 | 0.20 | -0.03 | 0.03 | 0.03 | -0.21 | -0.07 | 0.01 |

Table S4: Results from the multiple regression analyses using species-level traits as response variable and *minimum values of environmental variables* of the plots where the species occur as explanatory variables. Coefficients bellow each variable are standardized and indicate the relative contribution of this variable within each model. Significant models are highlighted in orange. See Table S3 for explanation of abbreviations of environmental variables.

|  | *R^2^* | *p*-value | Max T | Min T | Sum P | Elev | Soil moist | Solar rad | ET | Arid index |
| --- | --- | --- | --- | --- | --- | --- | --- | --- | --- | --- |
|  |  |  |  |  |  |  |  |  |  |  |
| Cold tolerance | 0.59 | <0.001 | -0.01 | 0.75 | 0.30 | <0.01 | -0.07 | 0.04 | -0.02 | -0.06 |
| Leaf N | 0.33 | <0.001 | 0.26 | -0.83 | -0.23 | -0.25 | 0.22 | -0.03 | 0.44 | 0.09 |
| Wood density | 0.32 | <0.001 | 0.54 | 0.24 | 0.21 | 0.05 | -0.40 | 0.12 | -0.38 | -0.08 |
| SLA | 0.27 | <0.001 | -0.19 | -0.40 | -0.21 | -0.04 | 0.22 | -0.18 | 0.17 | 0.19 |
| Waterlogging tolerance | 0.25 | <0.001 | 0.60 | -0.40 | -0.07 | -0.12 | 0.20 | 0.11 | -0.16 | -0.14 |
| Fire tolerance | 0.21 | <0.001 | -0.08 | -0.41 | -0.17 | -0.10 | -0.02 | 0.36 | -0.02 | 0.11 |
| Leaf P | 0.19 | 0.07 | 0.36 | -0.09 | -0.29 | -0.24 | 0.40 | 0.07 | -0.15 | 0.08 |
| Height | 0.18 | <0.001 | -0.24 | 0.24 | 0.01 | -0.01 | -0.28 | -0.13 | -0.16 | 0.08 |
| Drought tolerance | 0.16 | 0.003 | -0.08 | 0.30 | 0.30 | 0.19 | -0.31 | 0.08 | 0.01 | -0.26 |
| Life span | 0.12 | 0.01 | 0.04 | 0.33 | -0.04 | 0.26 | -0.20 | -0.07 | -0.12 | 0.07 |
| Seed mass | 0.10 | 0.04 | -0.15 | 0.09 | 0.25 | -0.11 | -0.19 | 0.47 | -0.19 | 0.06 |
| Leaf shape | 0.10 | 0.03 | -0.19 | -0.40 | -0.21 | -0.04 | 0.22 | -0.18 | 0.17 | 0.19 |
| Bark thickness | 0.08 | 0.19 | -0.07 | 0.13 | 0.07 | -0.11 | -0.25 | <0.01 | -0.06 | <0.01 |
| Winter buds size | 0.07 | 0.39 | -0.06 | 0.19 | -0.2 | -0.03 | 0.16 | -0.25 | -0.04 | 0.14 |
| Growth rate | 0.06 | 0.35 | 0.12 | -0.19 | -0.12 | -0.26 | 0.11 | -0.07 | 0.26 | -0.04 |
| Dispersal type | 0.06 | 0.23 | 0.16 | -0.28 | -0.21 | -0.03 | -0.07 | -0.19 | 0.17 | 0.11 |
| Shade tolerance | 0.05 | 0.50 | -0.14 | 0.14 | -0.12 | -0.07 | 0.19 | -0.06 | -0.13 | 0.06 |

Table S5: Results from the multiple regression analyses using species-level traits as response variable and *maximum values of environmental variables* of the plots where the species occur as explanatory variables. Coefficients bellow each variable are standardized and indicate the relative contribution of this variable within each model Significant models are highlighted in orange. See Table S6 for explanation of abbreviations of environmental variables.

|  | *R^2^* | *p*-value | Max T | Min T | Sum P | Elev | Soil moist | Solar rad | ET | Arid index |
| --- | --- | --- | --- | --- | --- | --- | --- | --- | --- | --- |
|  |  |  |  |  |  |  |  |  |  |  |
| Cold tolerance | 0.33 | <0.001 | -0.10 | 0.58 | -0.48 | 0.18 | 0.07 | -0.49 | -0.19 | -0.38 |
| SLA | 0.28 | <0.001 | -0.11 | 0.11 | 0.18 | 0.23 | <0.01 | -0.17 | -0.21 | 0.38 |
| Waterlogging tolerance | 0.27 | <0.001 | -0.25 | 0.14 | 0.21 | -0.22 | 0.68 | 0.25 | -0.29 | -0.39 |
| Fire tolerance | 0.18 | 0.001 | -0.18 | -0.10 | 0.07 | -0.29 | 0.14 | 0.50 | -0.07 | 0.27 |
| Drought tolerance | 0.16 | 0.007 | 0.18 | -0.03 | -0.11 | 0.14 | -0.42 | 0.08 | 0.45 | -0.25 |
| Wood density | 0.15 | 0.04 | 0.40 | -0.13 | 0.02 | <0.01 | -0.42 | 0.01 | 0.25 | <0.01 |
| Leaf N | 0.15 | 0.07 | 0.28 | -0.44 | 0.26 | -0.07 | 0.14 | -0.09 | <0.01 | -0.02 |
| Shade tolerance | 0.13 | 0.03 | 0.12 | 0.33 | -0.25 | 0.25 | -0.04 | -0.29 | -0.12 | 0.14 |
| Bark thickness | 0.11 | 0.04 | -0.13 | -0.03 | 0.55 | -0.35 | -0.38 | 0.52 | 0.23 | 0.07 |
| Dispersal mode | 0.10 | 0.02 | -0.28 | 0.06 | 0.11 | -0.20 | 0.36 | 0.34 | -0.12 | <0.01 |
| Leaf P | 0.10 | 0.5 | 0.26 | -0.44 | 0.37 | 0.30 | -0.31 | -0.20 | 0.04 | -0.16 |
| Life span | 0.07 | 0.21 | 0.10 | 0.09 | -0.07 | 0.11 | -0.16 | -0.02 | -0.10 | -0.01 |
| Height | 0.07 | 0.10 | -0.06 | 0.22 | 0.08 | -0.02 | 0.01 | 0.15 | -0.05 | 0.11 |
| Winter buds size | 0.06 | 0.55 | 0.05 | 0.16 | -0.16 | 0.23 | 0.16 | -0.29 | -0.11 | -0.06 |
| Seed mass | 0.06 | 0.29 | 0.31 | -0.08 | -0.22 | 0.16 | -0.05 | -0.22 | -0.07 | -0.01 |
| Leaf shape | 0.05 | 0.34 | -0.14 | 0.17 | -0.21 | -0.15 | 0.12 | 0.09 | 0.09 | 0.19 |
| Growth rate | 0.04 | 0.72 | 0.11 | -0.13 | 0.01 | 0.06 | 0.31 | <0.01 | -0.31 | -0.10 |
