# Supplementary material for: Stress from cold and drought as drivers of functional trait spectra in North American angiosperm tree assemblages
Source: Ecol Evol. 2017 Aug 14;7(18):7548–59. doi: 10.1002/ece3.3297 (PMC5606901; doi:10.1002/ece3.3297)

Appendix S7 – Partial dependence plots (response curves) for the 17 traits and species' environmental tolerances.

Partial dependence plots are used to graphically characterise relationships between individual predictor variables and predicted probabilities of the explanatory variables obtained from the random forest models. Partial dependence plots show the effect of a predictor variable after averaging out the effects of the other predictor variables in the model. Plots have been done using the function `*partialPlot*´ of the `*RandomForest*´ R package.

a) Cold tolerance

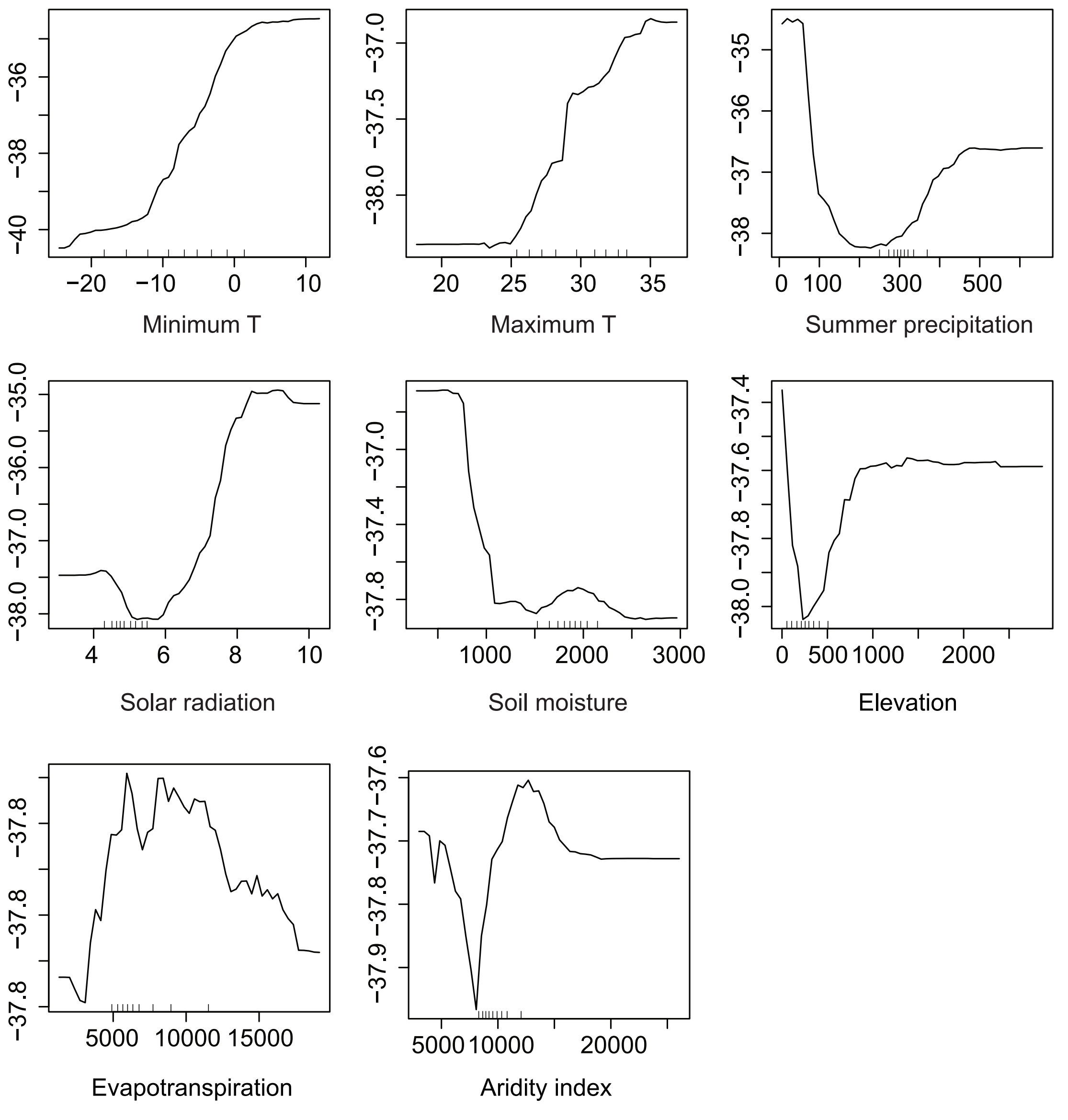

b) Drought tolerance

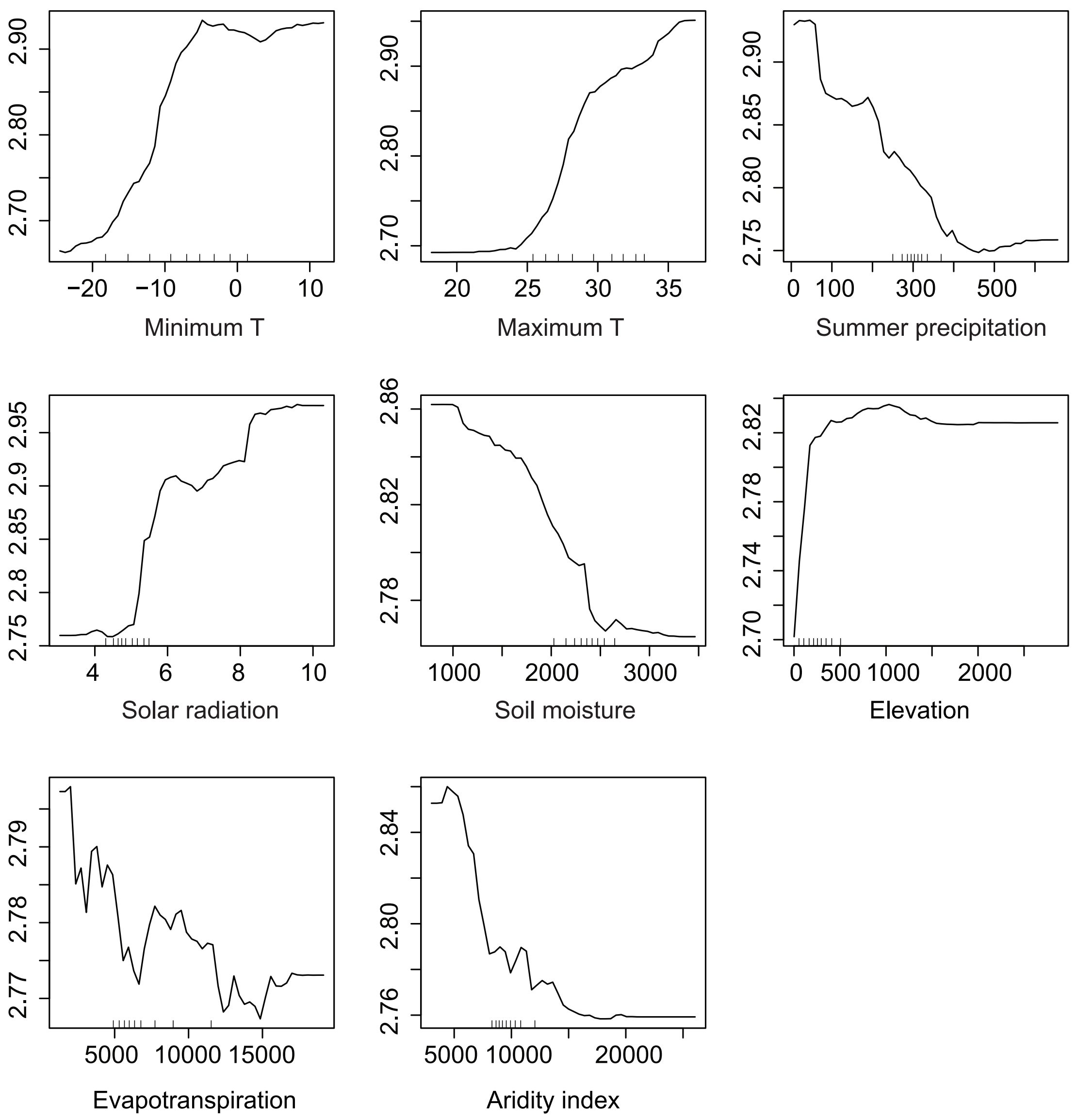

c) Shade tolerance

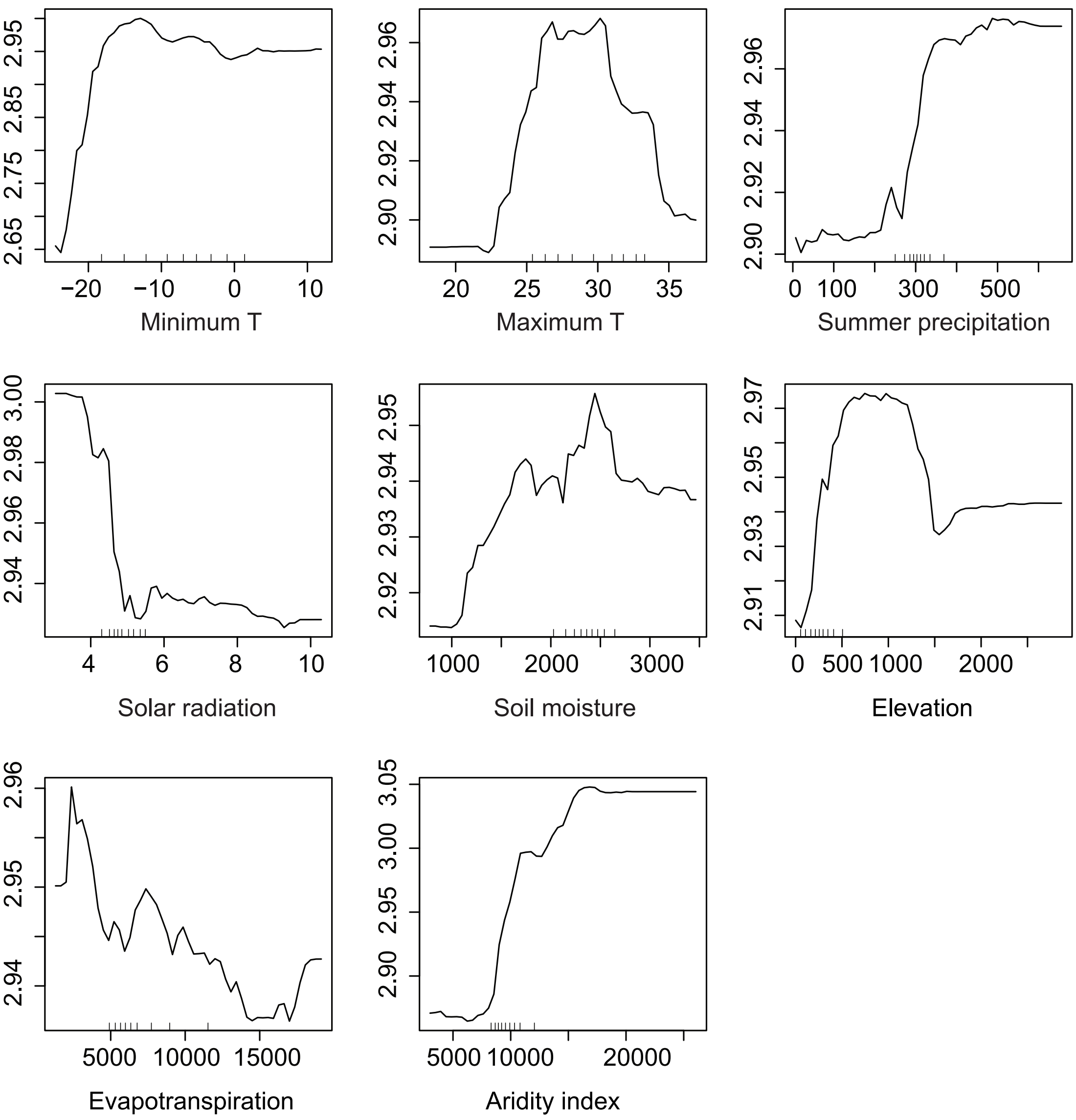

d) Waterlogging tolerance

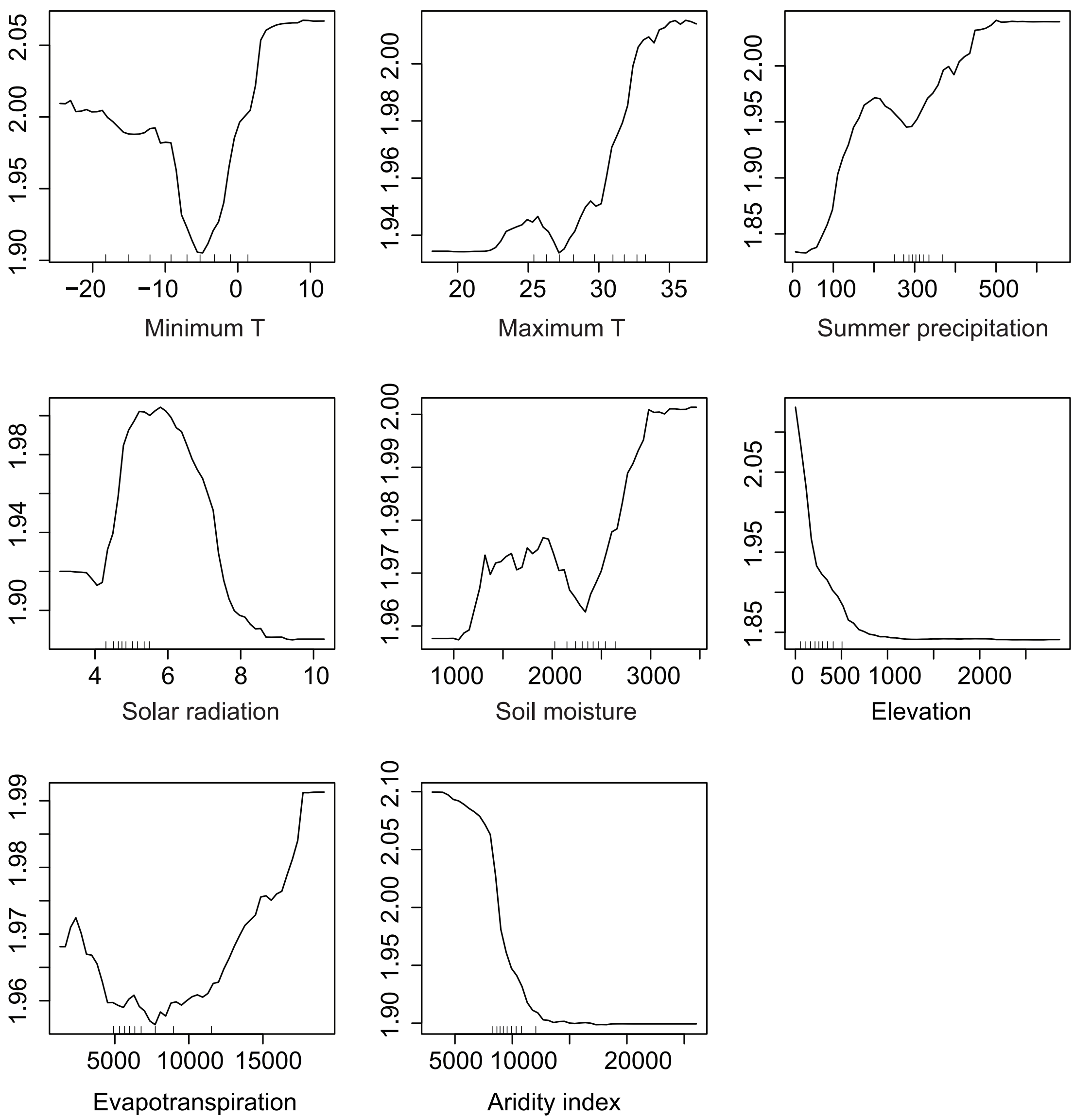

e) Fire tolerance

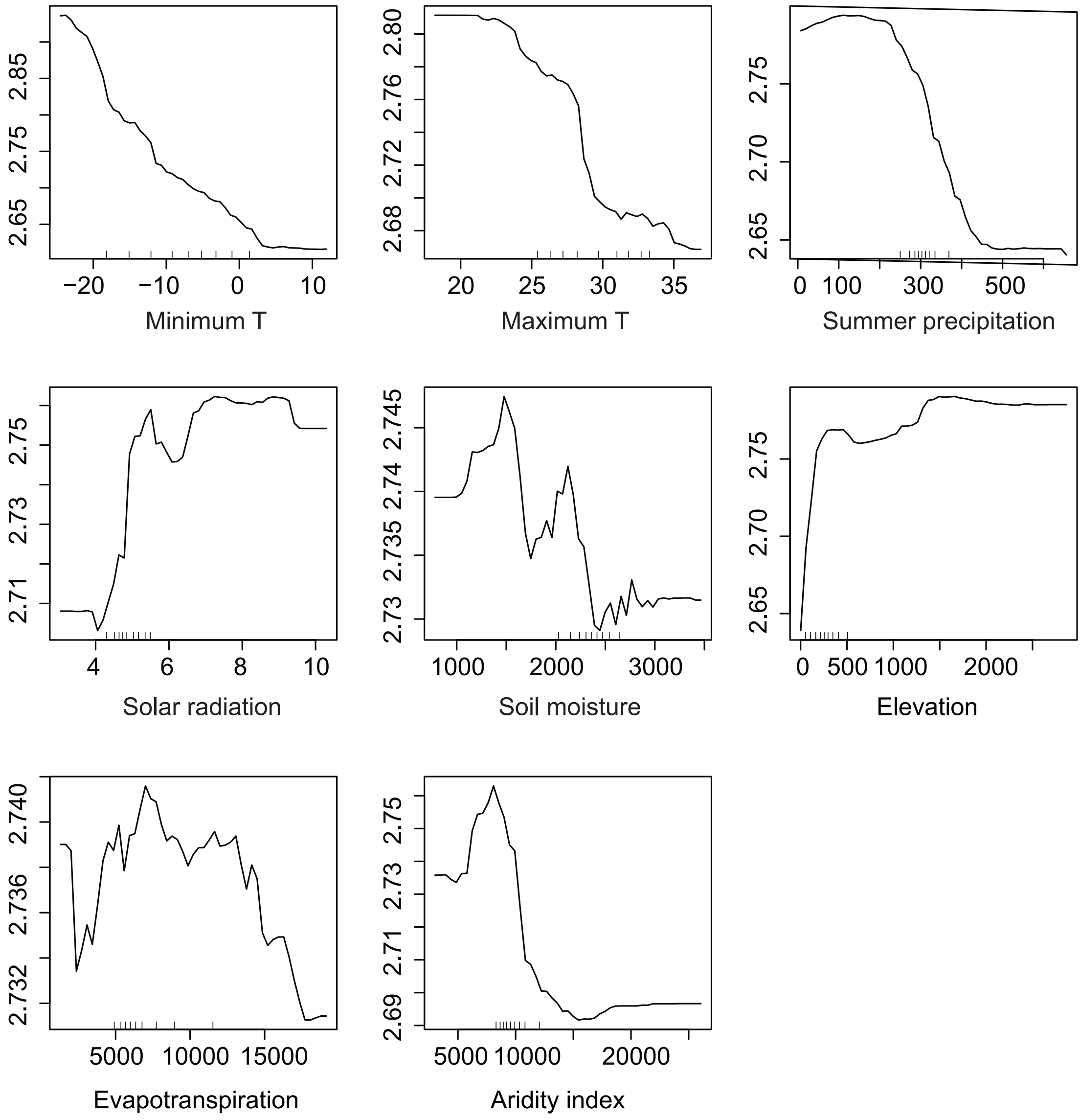

f) Growth rate

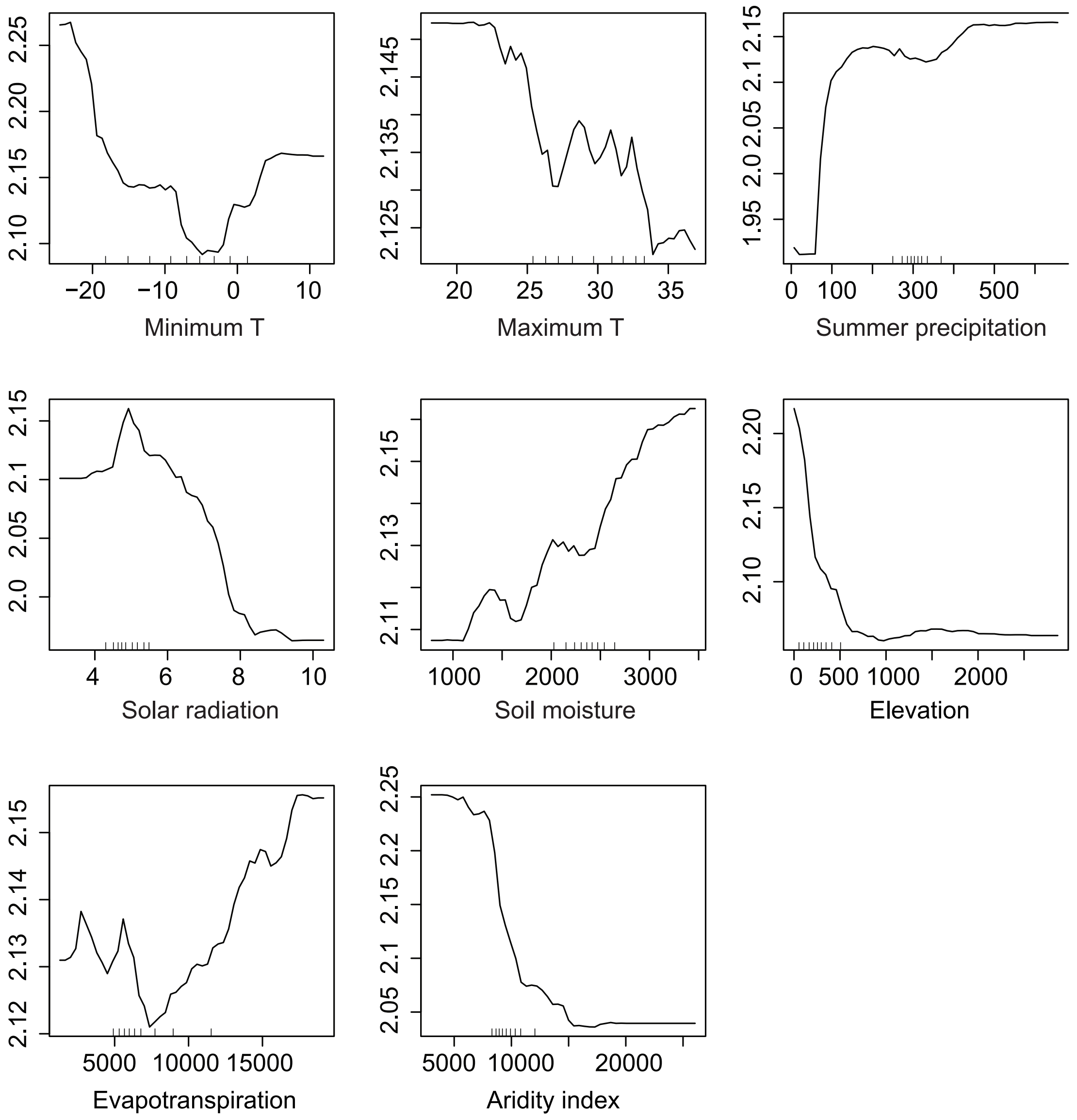

g) Seed size

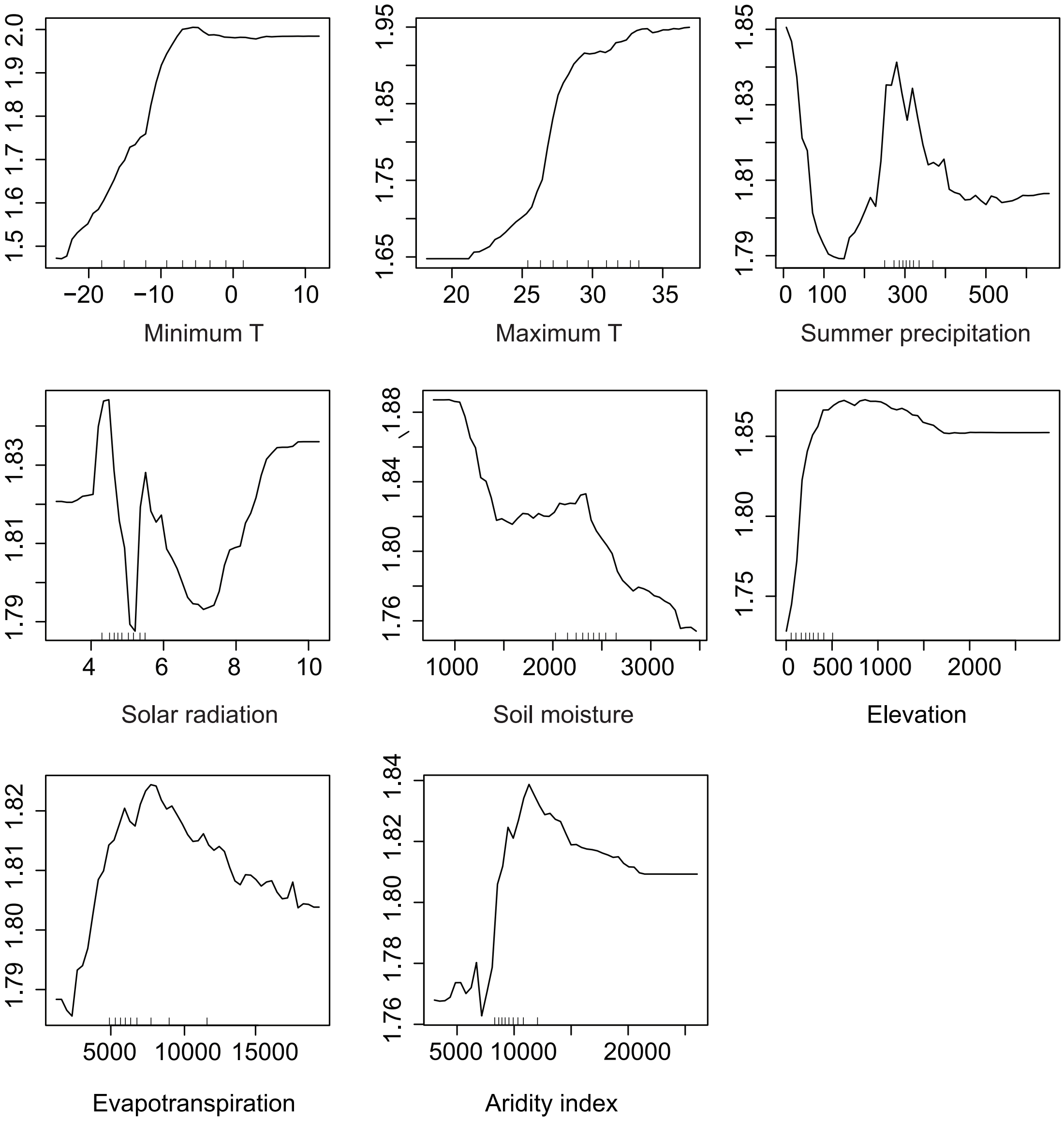

Minimum T
Maximum T
Summer precipitation
Solar radiation
Soil moisture
Elevation
Evapotranspiration
Aridity index

h) Dispersal mode

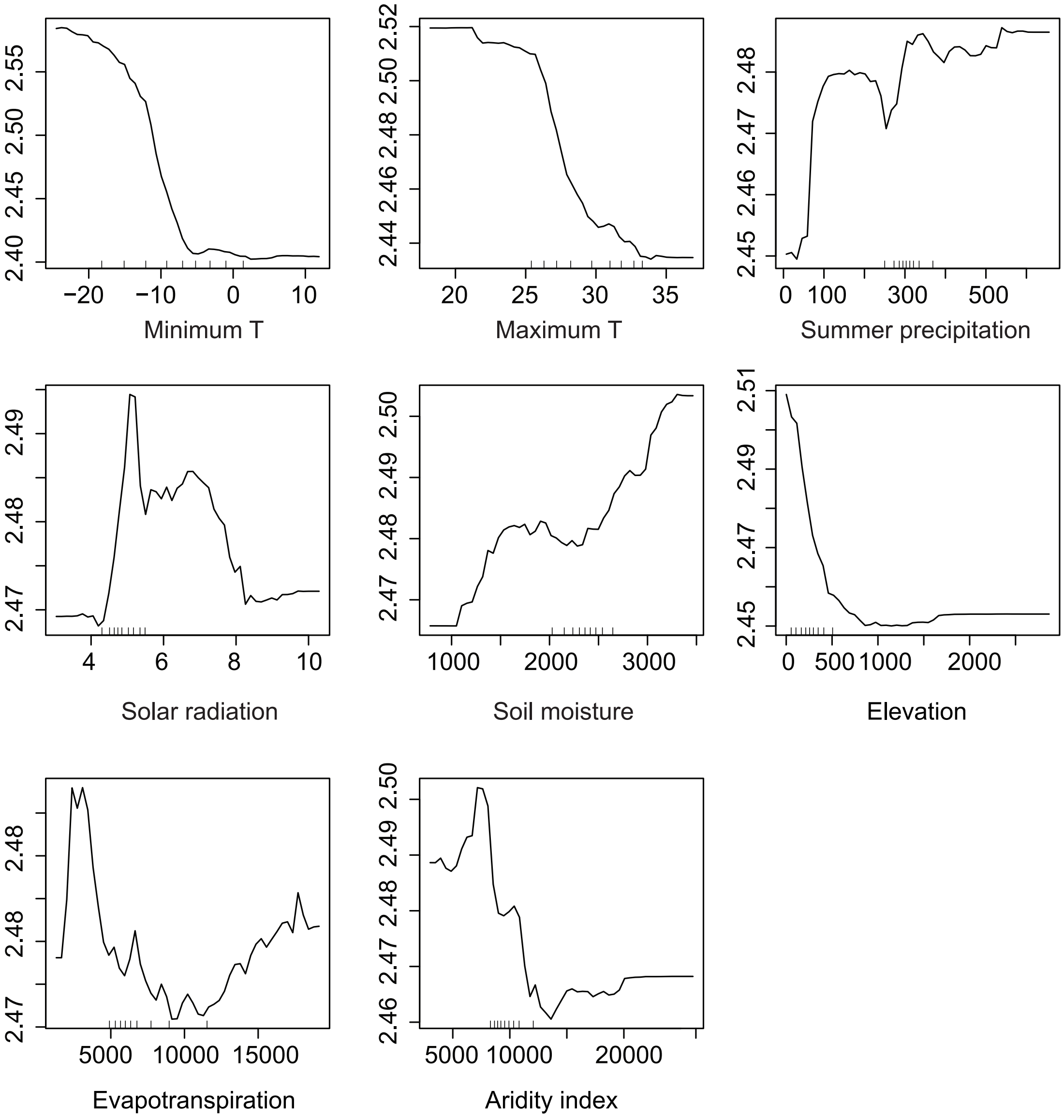

i) Wood density

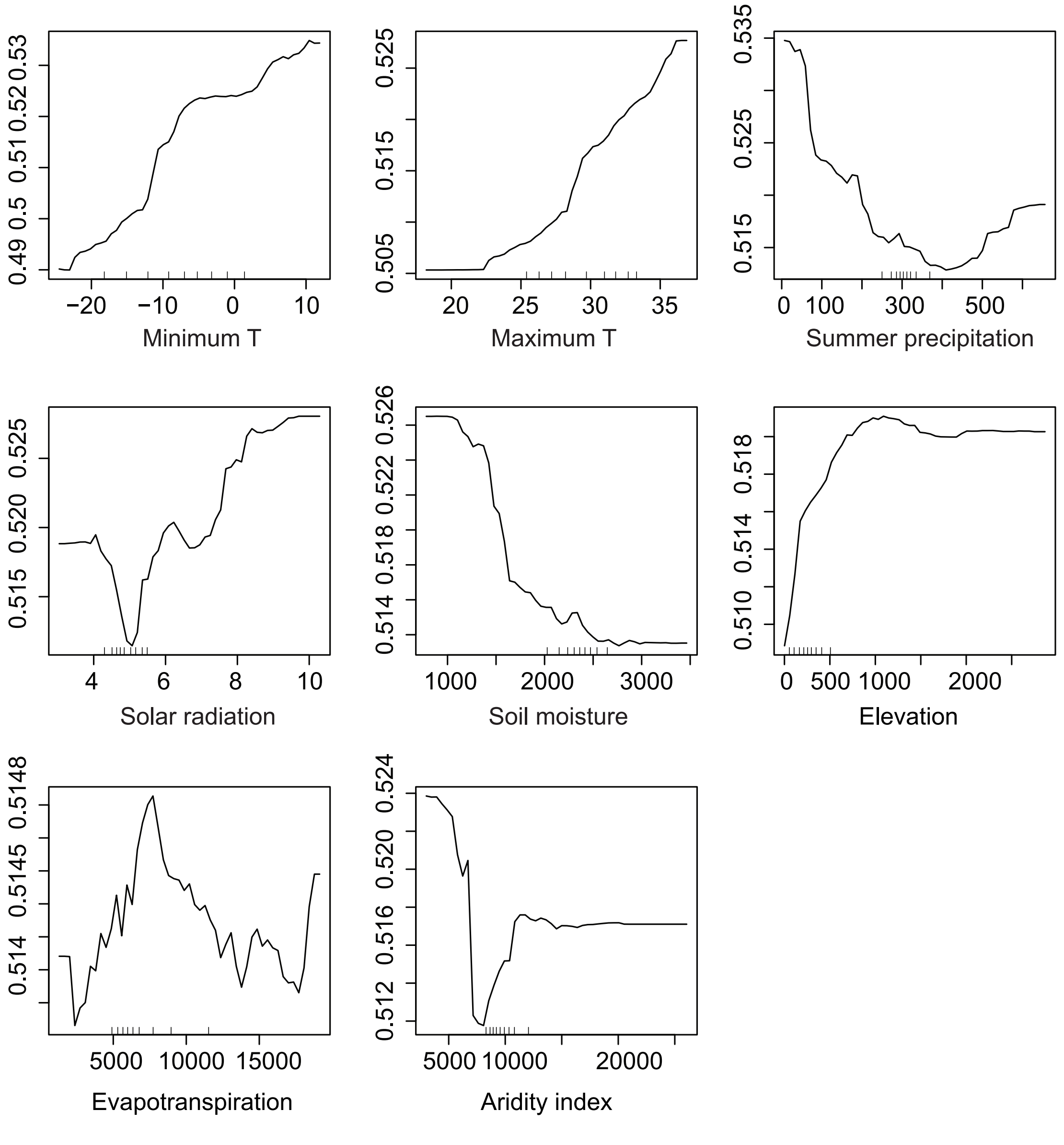

j) Winter buds size

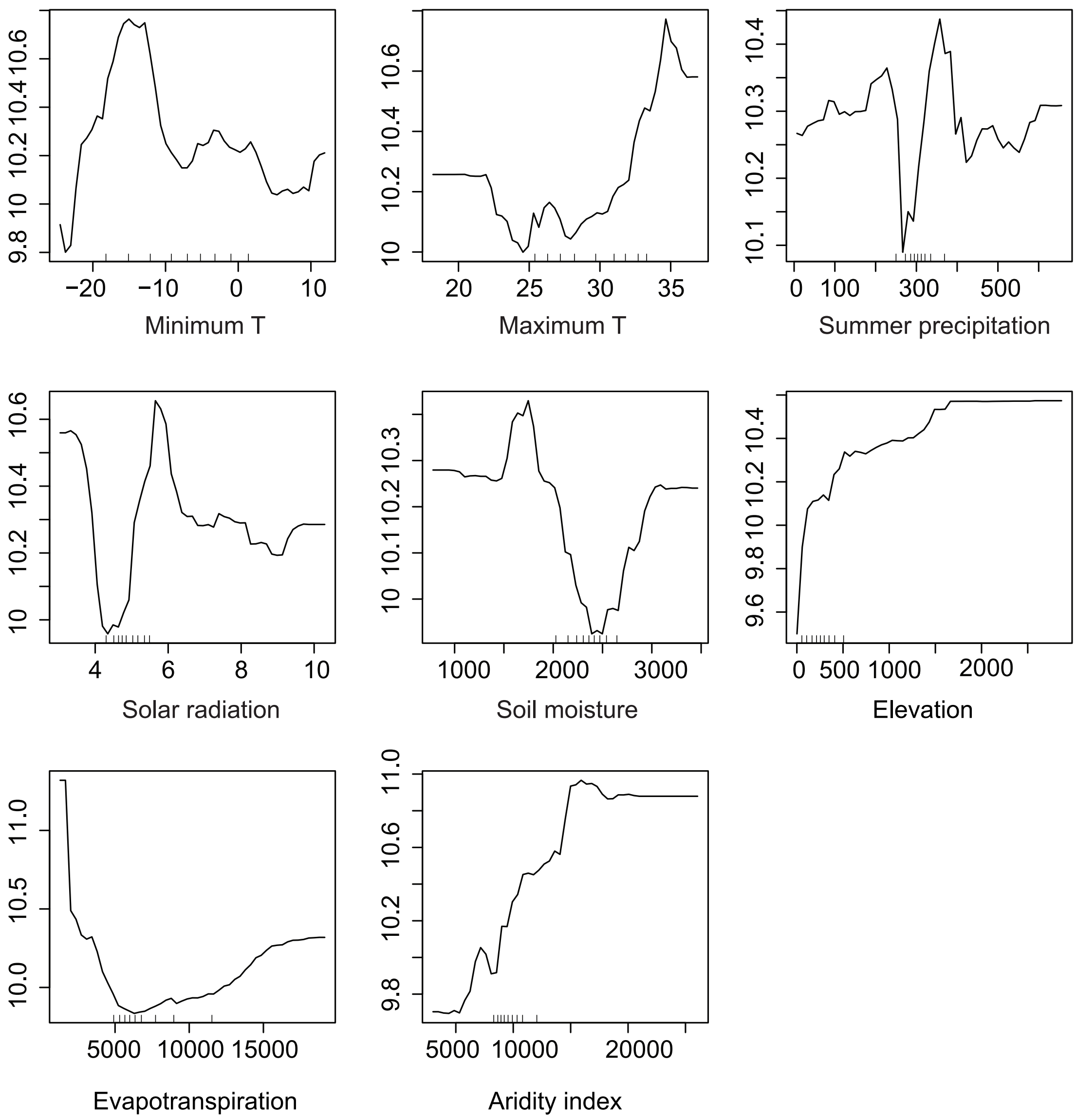

k) SLA

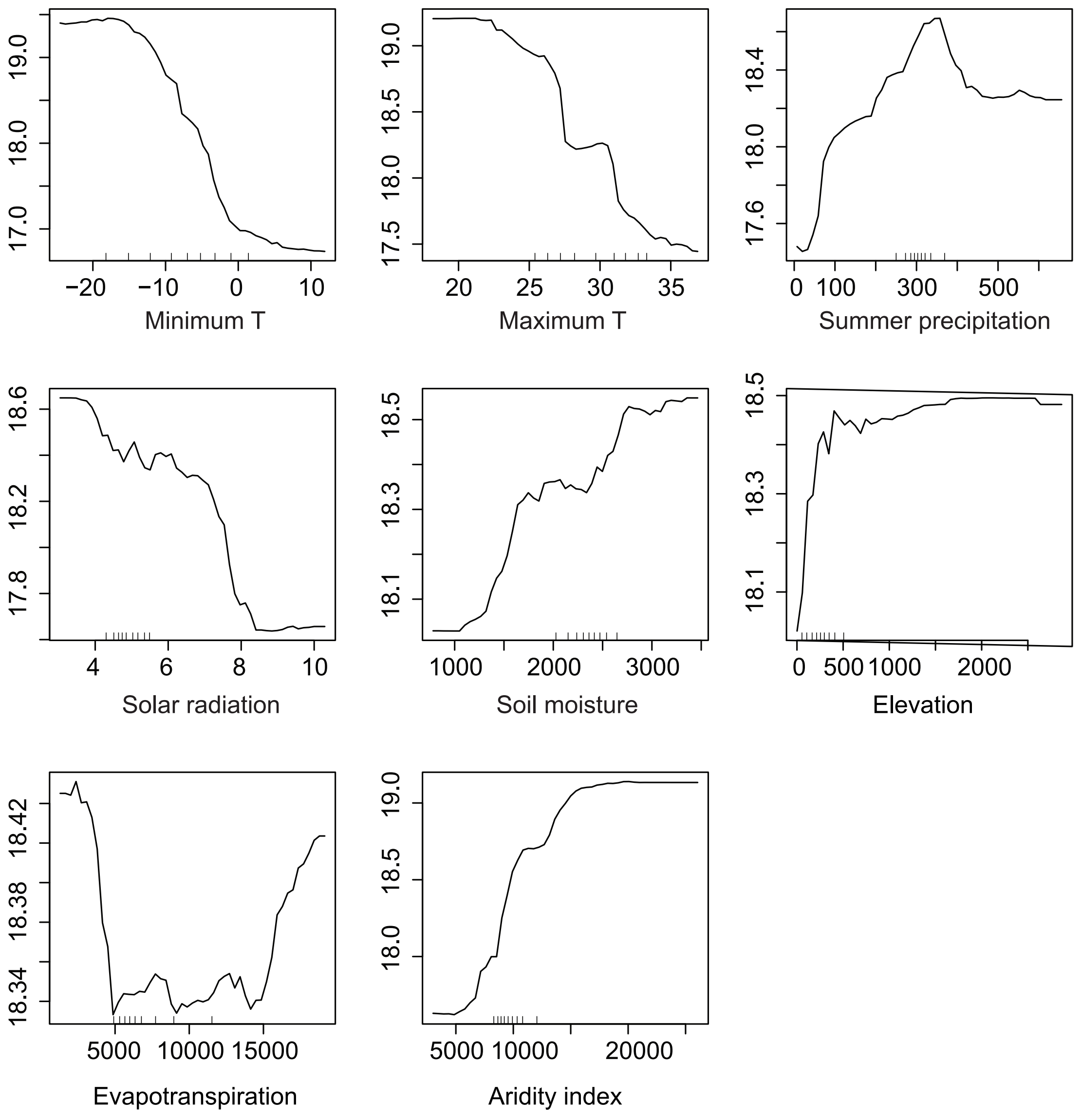
Minimum T
Maximum T
Summer precipitation
Solar radiation
Soil moisture
Elevation
Evapotranspiration
Aridity index

l) Life span

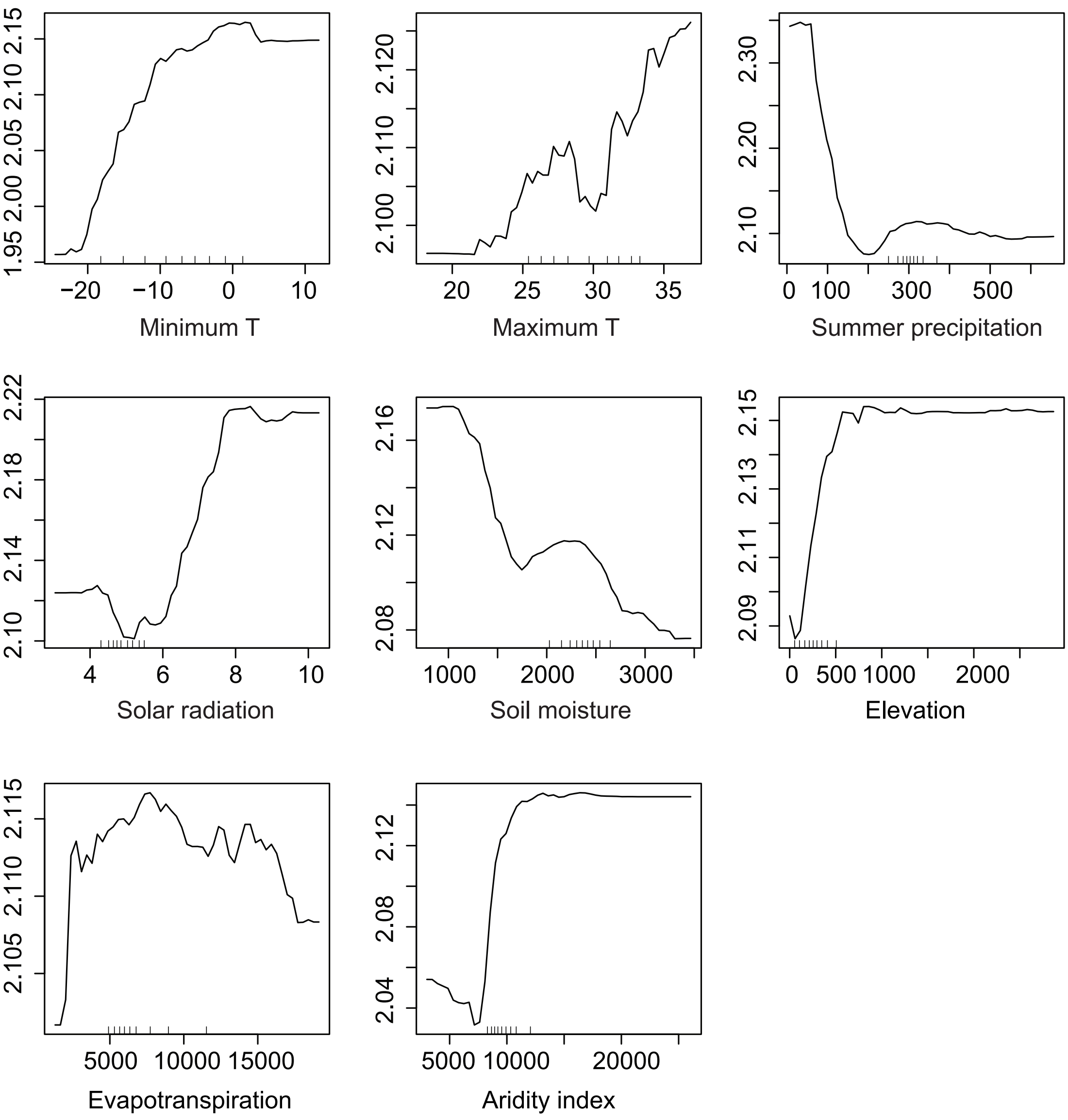
1.95 2.00 2.05 2.10 2.15
−20 −10 0 10
Minimum T
2.100 2.110 2.120
20 25 30 35
Maximum T
2.10 2.20 2.30
0 100 300 500
Summer precipitation
2.10 2.14 2.18 2.22
4 6 8 10
Solar radiation
2.08 2.12 2.16
1000 2000 3000
Soil moisture
2.09 2.11 2.13 2.15
0 500 1000 2000
Elevation
2.105 2.110 2.115
5000 10000 15000
Evapotranspiration
2.04 2.08 2.12
5000 10000 20000
Aridity index

m) Leaf shape

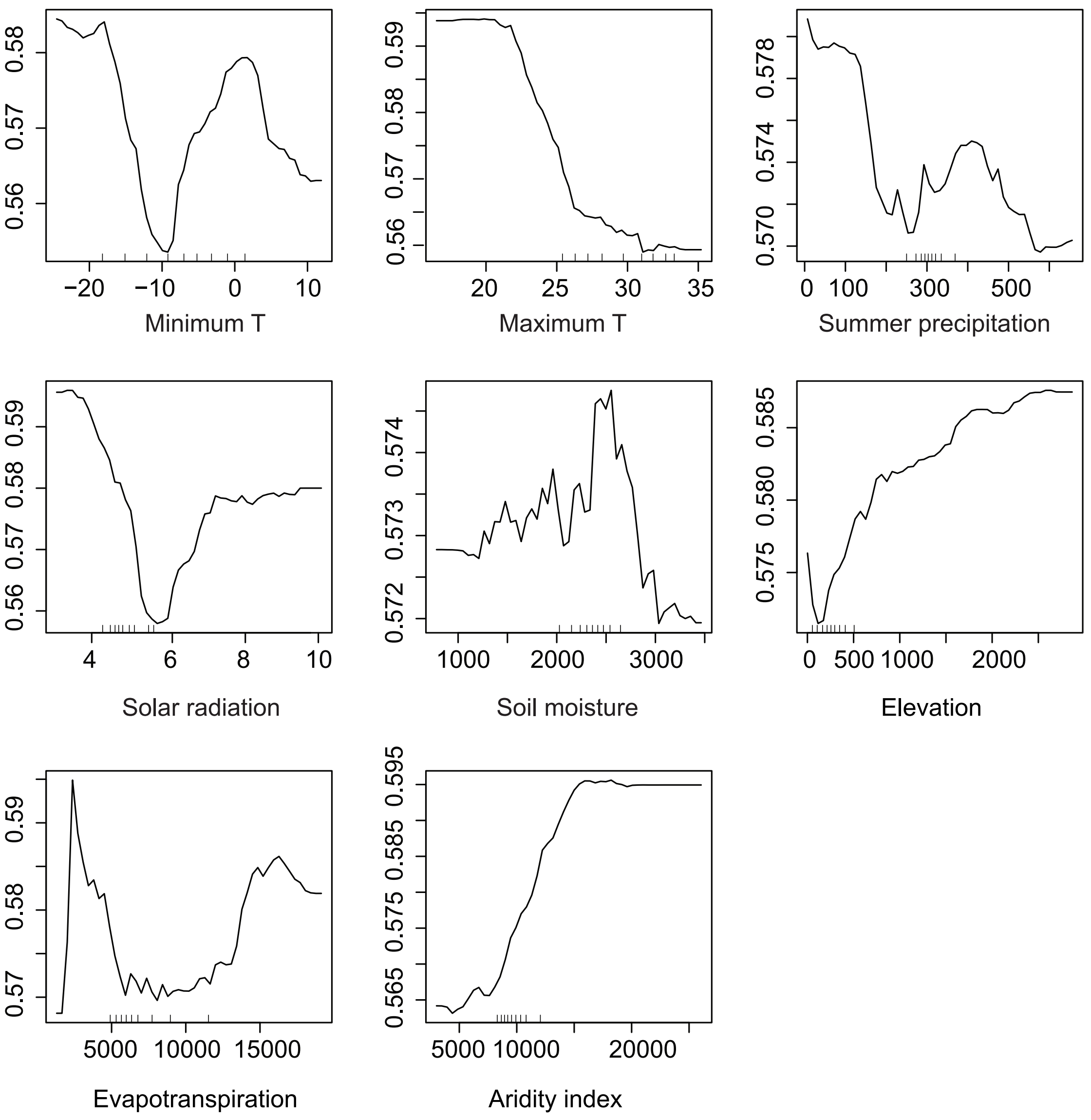

0.56
0.57
0.58
−20
−10
0
10
Minimum T
0.56
0.57
0.58
0.59
20
25
30
35
Maximum T
0.570
0.574
0.578
0
100
300
500
Summer precipitation
0.56
0.57
0.58
0.59
4
6
8
10
Solar radiation
0.572
0.573
0.574
1000
2000
3000
Soil moisture
0.575
0.580
0.585
0
500
1000
2000
Elevation
0.57
0.58
0.59
5000
10000
15000
Evapotranspiration
0.565
0.575
0.585
0.595
5000
10000
20000
Aridity index

n) Leaf N content

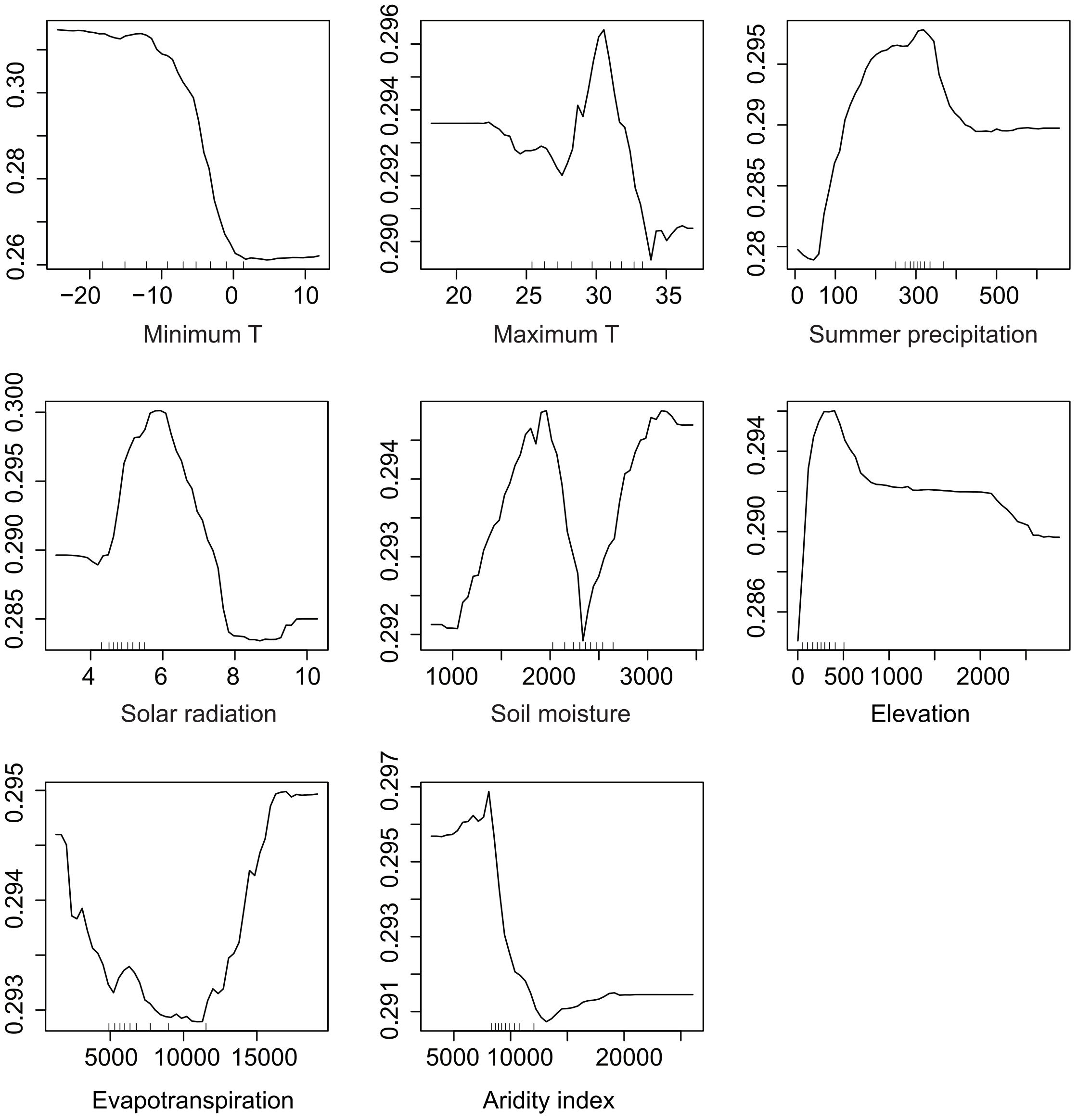

o) Leaf P content

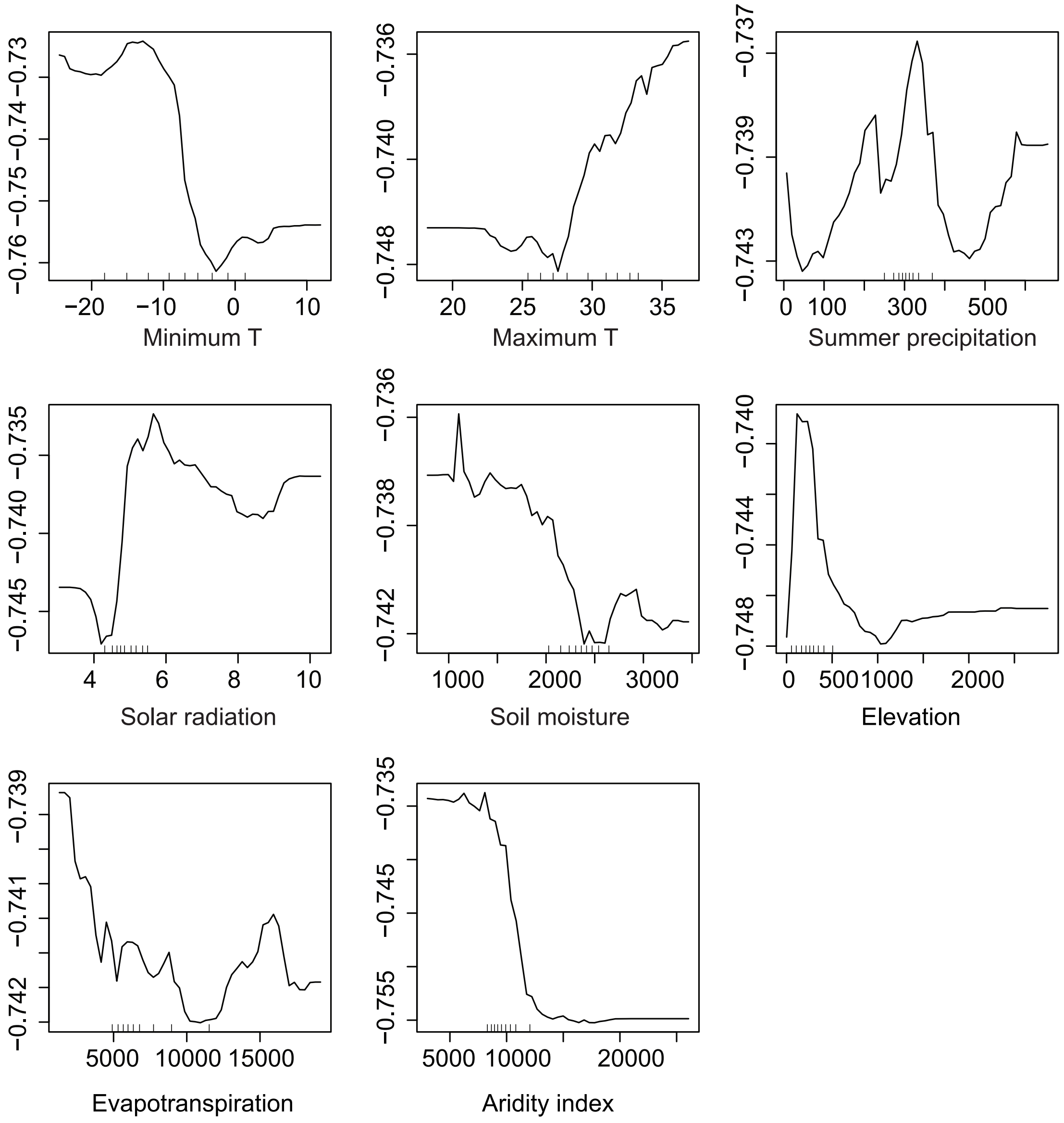

p) Tree height

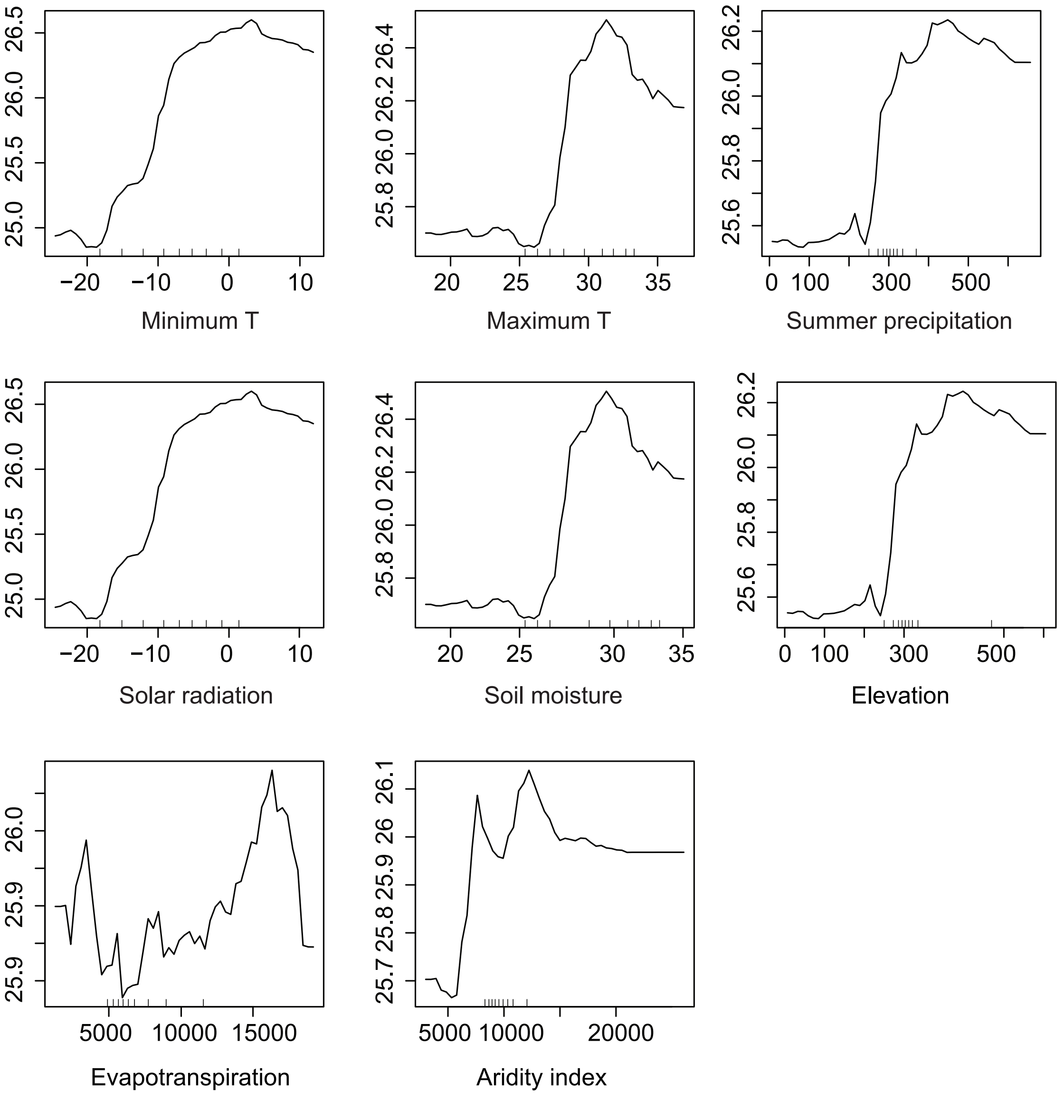

r) Bark thickness

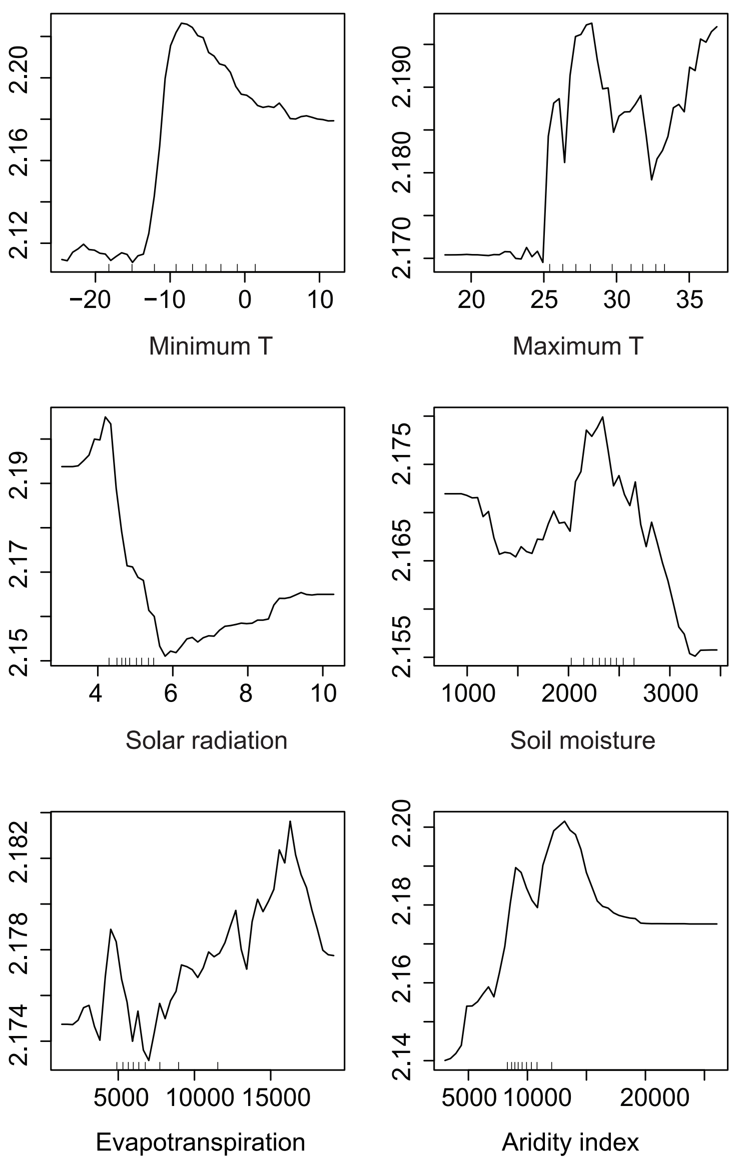

Supplement: Supplementary file 4 [file ECE3-7-7548-s004.pdf]
